# Pleiotropic SNPs and genes linked to enhanced seed longevity and vigor for climate resilient pearl millet production

**DOI:** 10.3389/fpls.2026.1717105

**Published:** 2026-03-31

**Authors:** Netyam Kannababu, Sandeep Nanjundappa, Renuka Malipatil, Bharathi Chunduri, Arutla Srikanth, Ronda Venkateswarlu, Jinu Jacob, Vijayakumar M. Malathi, Tara Chellapilla Satyavathi, Nepolean Thirunavukkarasu

**Affiliations:** 1 Seed Science and Technology Lab, ICAR-Indian Institute of Millets Research, Hyderabad, India; 2 Genomics and Molecular Breeding Lab, Global Centre of Excellence on Millets (Shree Anna), ICAR-Indian Institute of Millets Research, Hyderabad, India; 3 Biochemistry Lab, ICAR-Indian Institute of Millets Research, Hyderabad, India; 4 Biotechnology Lab, ICAR-Indian Institute of Millets Research, Hyderabad, India

**Keywords:** pearl millet, accelerated aging, genome-wide association study, marker-trait association, climate resilience

## Abstract

Seed longevity and vigor are critical factors for sustainable crop production, particularly in environments prone to stress, where consistent germination and seedling establishment are crucial. This study investigates the genetic architecture underlying seed longevity and vigor traits in pearl millet (*Pennisetum glaucum* [L.] R. Br.) through genome-wide association studies (GWAS). The GWAS panel comprising 201 inbred lines representing B and R lines was phenotyped under both control and accelerated aging (AA) conditions for 27 seed longevity and seedling vigor traits and their relative measures. Phenotypic analysis revealed significant changes in seed traits following AA in germination percentages, seedling vigor indices and mean germination times. A 4K mid-density SNP panel was used for genotyping, producing 2,015 high-confidence SNPs after stringent filtering for GWAS. The Bayesian information and linkage disequilibrium iteratively nested keyway (BLINK) and Multi-locus mixed model (MLMM) models identified 413 significant marker-trait associations (MTAs), with 185 MTAs common to both models. A pleiotropic SNP, *PMSnpB394* on chromosome 2, was linked to 14 seed longevity traits. Key genes associated with these traits include hormone signaling (*Auxin response factor, DOG1*), stress response (*LEA_2, 10 kDa heat shock protein*), metabolism (*Glyceraldehyde-3-phosphate dehydrogenase, Beta-galactosidase*) and seed coat structure (*Peroxidase, 3-Ketoacyl-CoA synthase*). These MTAs and candidate genes provide valuable insights into the molecular mechanisms underlying seed longevity and vigor and serve as promising targets for marker-assisted selection and breeding strategies aimed at developing climate-resilient cultivars in the rainfed ecologies.

## 1 Introduction

In the face of global climate change, ensuring crop productivity and sustainability has become a challenge for agricultural practices worldwide (Malhi et al., 2021). Among the diverse cereal crops cultivated globally, pearl millet (*Pennisetum glaucum* [L.] R. Br.) stands out for its adaptability to drought and nutrient-poor soils, making it a vital crop for ensuring food security and sustaining livelihoods in the arid and semi-arid regions of Africa and Asia (Satyavathi et al., 2021). At the heart of sustainable crop production lies the ability of seeds to maintain viability and vigor under adverse climatic conditions, which is crucial for long-term adaptability and productivity (Reed et al., 2022). Seed aging during storage results in a significant decline in quality, potentially leading to losses of up to 25% (McDonald, 1999; Rajjou et al., 2008; Schwember and Bradford, 2010). Even under optimal storage conditions, aging can induce genetic damage and reduce integrity, posing germplasm conservation challenges and potentially impacting crop yields (Perez-Garcia et al., 2008; Singh et al., 2022). However, the genetic mechanisms underlying seed vigor and longevity remain elusive.

Seed vigor is a key determinant of crop establishment, ensuring uniform emergence, rapid growth and resilience under diverse field conditions (Rajjou et al., 2012; Ventura et al., 2012). Seed vigor gradually declines with aging, making seeds more sensitive to stress during sowing, which in turn leads to poor emergence and establishment and ultimately reduces crop productivity. Various factors influence seed vigor, including genetic, environmental and storage factors, which directly regulate the degree of expression of seed vigor (Sun et al., 2007). Substantial variation for seed vigor is reported both among crop species and among cultivars within the same species (Yu et al., 1999; Tang and Ma, 2007; Cheng et al., 2003; Oluwaranti et al., 2020). Extended storage under traditional conditions can diminish seed viability, reduce germination rates and ultimately lower crop yields. The longevity combined with the vigor of seeds is crucial for crop sustainability, especially as farmers increasingly rely on stored seeds due to the high cost of acquiring fresh seeds. The impact of global warming further exacerbates these issues, making seed longevity a critical concern for farmers who depend on farm-saved seeds (Rehmani et al., 2023).

During dry storage, seed viability decreases gradually due to aging processes, with initial symptoms manifesting as delayed germination and poor seedling establishment, eventually leading to complete loss of viability and reduced crop yield (Seshu et al., 1988; Ghassemi-Golezani et al., 2010). Pearl millet endosperm is rich in fatty acids, which play a critical role in determining seed longevity. During storage or aging, the oxidation of membrane lipids leads to the generation of reactive oxygen species, causing cellular damage and reduced vigor (Feng et al., 2017). These changes pose significant challenges to pearl millet productivity and the effectiveness of seed storage programs, threatening food security in rainfed farming systems. As the study of seed longevity under natural, ambient storage conditions requires a long time for ageing, several artificial ageing methods are used to understand the trait genetics. One of the most rapid methods is the accelerated aging (AA) test. AA test effectively simulates the molecular and biochemical processes associated with natural seed aging, enabling a more rapid and accurate assessment of seed longevity (McDonald, 1999).

Environmental factors influence seed longevity during seed formation, harvest and storage. Studies have shown that humidity and temperature during storage are crucial in determining seed longevity (Zhou et al., 2020). In rice, aging is associated with decreased germination rates and poor seedling establishment, which negatively affects overall plant growth and productivity (Zhou et al., 2024). Similarly, in sorghum, aged seeds often exhibit reduced growth rates and increased susceptibility to fungal pathogens, further compromising crop performance (Beyene et al., 2024).

Seed vigor and longevity are complex quantitative traits governed by multiple genes with minor effects, often influenced by environmental interactions (Kannababu et al., 2025). Genome-wide association studies (GWAS) exploit natural allelic diversity present in diverse germplasm panels, enabling the detection of multiple loci associated with quantitative traits. GWAS serves as a powerful approach for dissecting the genetic architecture (Nepolean et al., 2013, 2014) of seed vigor and longevity, facilitating the identification of key genomic regions and candidate genes linked to seed vigor and longevity.

QTL and genes associated with seed longevity were identified across various crops, highlighting the genetic basis of this complex trait (Pirredda et al., 2024). A GWAS study of 270 Arabidopsis genotypes identified genomic regions linked to seed longevity under experimental and natural aging. Positive (*PSAD1, CYP86A8, MYB47*) and negative (*RBOHD, KNAT7*) genes for seed longevity were found, with *CYP86A8, MYB47, KNAT7* and *SEP3* contributing to seed coat protection (Renard et al., 2020a). In wheat, GWAS study identified 23 loci linked to seed longevity under artificial aging from 166 recombinant inbred lines (Zuo et al., 2019). Candidate genes for these loci suggested the role of ABA signal transduction and stress resistance, including a *FAR1-related protein* and *delta-1-pyrroline-5-carboxylate synthase*, in abiotic stress responses and proline metabolism.

In pearl millet, there is limited research on the molecular studies and genetic architecture of seed longevity and seedling vigor. The present study uses a GWAS approach to investigate the genetic architecture of seed traits associated with vigor and longevity in pearl millet. By integrating phenotypic data from controlled accelerated aging treatment with mid-density SNP genotyping, we pursued to uncover marker-trait associations (MTAs) related to seed longevity and vigor. Our findings elucidated the genetic basis of seed longevity and seedling vigor and identified key genomic regions for breeding and gene-editing programs for enhancing seed storage resilience and extending longevity under changing climatic conditions. This will contribute to more resilient seeds and strengthen food security in regions where pearl millet is a staple crop.

## 2 Materials and methods

### 2.1 Association mapping panel

A set of 201 genetically diverse panel of pearl millet inbreds, representing two heterotic pools, B (maintainer) and R (restorer) from the pearl millet breeding program at ICAR-Indian Institute of Millet Research, Hyderabad was used in this experiment. The panel was carefully selected to capture the maximum genetic diversity present in the IIMR breeding program. Detailed information on these inbreds is provided in **Supplementary Table S1**.

### 2.2 Phenotyping

The GWAS panel was phenotyped for 27 seed traits under two experimental conditions: control and accelerated aging (AA) treatment. The evaluated traits include seed germination (%), germination index, germination rate index, mean germination time (days), root length (cm), shoot length (cm), seedling vigor index (1), seedling dry weight (mg) and seedling vigor index (2). For each trait, measurements were taken for both the control, AA treatments and their relative values, with the experiment conducted in three replications. AA test conditions involved a 6-day exposure as per prior standardization for pearl millet genotypes in an accelerated aging chamber (*Memmert-HPP 108/749, Germany*) maintained at 90% or higher humidity and a temperature of 44 ± 1°C. Both control (fresh) seeds and AA-treated seeds from the 201 accessions were evaluated for seed quality traits according to standard seed germination protocols (ISTA, 2021).

Germination percentage (G) represents the total number of seedlings at the end of the test on the 8^th^ day. Germination index (GI) was calculated as GI = (8 × n_1_) + (7 × n_2_) +… (1 × n_8_). Where, n1, n2 …. n8 is the number of germinated seeds on the first, second and subsequent days until the 8^th^ day; 8, 7… and 1 are the corresponding weights assigned to the number of germinated seeds on each day (Kader, 2005). Germination rate index (GRI) was determined using the formula GRI = G_1_/1 + G_2_/2 + G_3_/3 +…. + G_n_/n (%/day), where G_1_ represents the germination percentage × 100 on the first day after sowing, G_2_ for the second day, and so on until the eighth day; 1, 2, …, and n represent the respective days of germination count (Esechie, 1994). The mean germination time (MGT) was calculated as MGT = ∑ n.D/n (days) (Ellis and Roberts, 1981), where n is the number of seeds germinated on day D. Mean root length (RL) (cm) of five normal seedlings was measured from the collar region to the tip of the primary root. Similarly, mean shoot length (SL) (cm) of five normal seedlings was measured from the collar region to the tip of the first leaf. Seedling dry weight (SDW) (mg) was recorded after drying 10 normal seedlings in a hot air oven at 80°C for 24 h. After drying, seedlings were cooled in a desiccator for 30 mins, and the mean dry weight of the seedlings was then measured. Seedling vigor index 1 (SVI1) was calculated by multiplying the final germination percentage by the mean seedling length (root + shoot), while seedling vigor index 2 (SVI2) was computed by multiplying the mean germination percentage by the mean seedling dry weight (SVI2).

Quantitative traits analyzed under both control and accelerated aging conditions encompass a range of germination and growth parameters. Initial germination under control conditions (GC, %) and after accelerated aging (GAA, %) were recorded, with relative germination after accelerated aging (GAAR = GAA/GC × 100, %). Germination indices under control (GIC) and accelerated aging (GIAA) conditions were assessed, along with their relative values (GIAAR = GIAA/GIC × 100, %). Germination rate index (GRIC for control and GRIAA for accelerated aging, %/day) and their relative performance (GRIAAR = GRIAA/GRIC × 100, %) were evaluated. Mean germination time under control (MGTC, days) and accelerated aging (MGTAA, days) was recorded, with relative mean germination time (MGTAAR = MGTAA/MGTC × 100, %) calculated to assess the impact of aging.

Root length measurements under control (RLC, cm) and accelerated aging (RLAA, cm) were taken, with relative root length (RLAAR = RLAA/RLC × 100, %) providing insight into root growth resilience. Similarly, shoot length under control (SLC, cm) and accelerated aging (SLAA, cm) was measured, and relative shoot length (SLAAR = SLAA/SLC × 100, %) was determined. Seedling vigor indices under both conditions were comprehensively evaluated: Seedling Vigor Index-1 for control (SVI1C = GC × (RL + SL)) and after accelerated aging (SVI1AA = GAA × (RL + SL)), with relative seedling vigor index-1 calculated as (SVI1AA/SVI1C × 100, %). Seedling dry weight under control (SDWC, mg) and accelerated aging (SDWAA, mg) conditions was measured and relative seedling dry weight (SDWAAR = SDWAA/SDWC × 100, %) was determined. Seedling Vigor Index-2 for control (SVI2C = GC × SDW) and after accelerated aging (SVI2AA = GAA × SDW) were calculated, with their relative index (SVI2AAR = SVI2AA/SVI2C × 100, %).

### 2.3 Genotyping

#### 2.3.1 Genomic DNA isolation

Genomic DNA was extracted from 10- to 15 seed samples of the GWAS panel inbreds, using the CTAB (Cetyl Trimethyl Ammonium Bromide) method as described by Murray and Thompson (1980), with modifications. DNA quantification was performed using both a spectrophotometer and a nanodrop 1000 (Thermo Scientific), with absorbance measurements at 260 and 280 nm used to assess DNA quality and quantity. Qualitative analysis was conducted by agarose gel electrophoresis, with the DNA samples resolved on a 1% agarose gel at 80V for 30 minutes. Post-electrophoresis, the bands were visualized and documented using a gel documentation system to check for RNA and protein contamination. Genotyping of the GWAS panel was carried out using a 4K SNP mid-density panel (Semalaiyappan et al., 2023).

#### 2.3.2 SNP quality control/SNP curation

Raw dataset containing 4,072 SNPs across 201 pearl millet genotypes was subjected to stringent filtering. Monomorphic SNPs were removed and only biallelic SNPs were retained. Additionally, SNPs with more than 15% heterozygosity, more than 20% missing data and a minor allele frequency less than 5% were excluded. After applying these filters, a final set of 2,015 high-confidence SNPs, distributed across seven chromosomes, was selected for GWAS.

### 2.4 Neighbor-joining tree and kinship analysis

The filtered genotype matrix was converted into a genetic distance matrix using the *dist.gene()* function implemented in the *“ape”* (Analyses of Phylogenetics and Evolution) package (Paradis et al., 2004) in R. Based on this distance matrix, a neighbor-joining (NJ) tree was constructed using the *nj()* function. The resulting NJ tree was visualized using the Interactive Tree of Life (iTOL) web tool (Letunic and Bork, 2021) to depict the genetic relationships among the pearl millet genotypes. The kinship matrix (K-matrix) was computed using the VanRaden method implemented in Genome Association and Prediction Integrated Tool (GAPIT v.3) (Wang and Zhang, 2021) to estimate pairwise genetic relatedness among genotypes.

### 2.5 Phenotypic data analysis

The adjusted means of replicates were obtained by fitting mixed linear models (MLM) and calculating best linear unbiased predictions (BLUPs), treating replication and genotype as random effects. BLUP for each genotype, along with analysis of variance (ANOVA), were estimated using the R package *“lme4”* (Bates et al., 2015). The BLUPs were derived using the following model (Henderson, 1975):


$$Y_{ik}=\;\mu +R_{i\;}+G_{k}+\;\epsilon_{ik}\;$$

Where, *Y_ik_* represents the trait of interest; μ is the overall mean effect; *R_i_* is the effect of the i^th^ replicate; *G_k_* is the effect of the k^th^ genotype; ϵ_ik_ is the error associated with the i^th^ replication and the k^th^ genotype, which is assumed to be normally and independently distributed, with mean zero and homoscedastic variance σ^2^. Broad sense heritability (*h^2^*) was estimated using the formula.


$$\;\;\;\;h^{2}=\;\frac{\sigma_{g}^{2}}{\sigma_{g}^{2}+\;\sigma_{e}^{2}\;/nreps}$$

Where, *σ^2^g* is the genotypic variance, *σ^2^e* is the error variance and *nreps* is the number of replications.

### 2.6 Genome-wide association mapping

GWAS was performed using the filtered set of 2,015 SNPs and BLUPs for each trait, employing the “MLMM” (Multiple Loci Mixed Model) and “BLINK” (Bayesian-information and Linkage-disequilibrium Iteratively Nested Keyway) models available in (GAPIT) v.3 (Wang and Zhang, 2021), with kinship and 15 principal components (PCs) as covariates. The MLMM model (Segura et al., 2012) enhances detection power and reduces false positives by incorporating multiple loci into the mixed model. BLINK model (Huang et al., 2019) effectively reduces false positives, allowing for identifying true marker-trait associations (MTAs). The quality of model fit was evaluated using Q-Q plots, which compared the expected versus observed −log10(p) values. Significant MTAs were identified using a *p*-value cut-off of 0.005. Circular manhatton plots were generated using Circos v0.69-9 (Krzywinski et al., 2009) to visualize the MTAs.

## 3 Results

### 3.1 Genetic variability and phenotypic distribution of seed vigor and longevity traits

**Figure 1:**
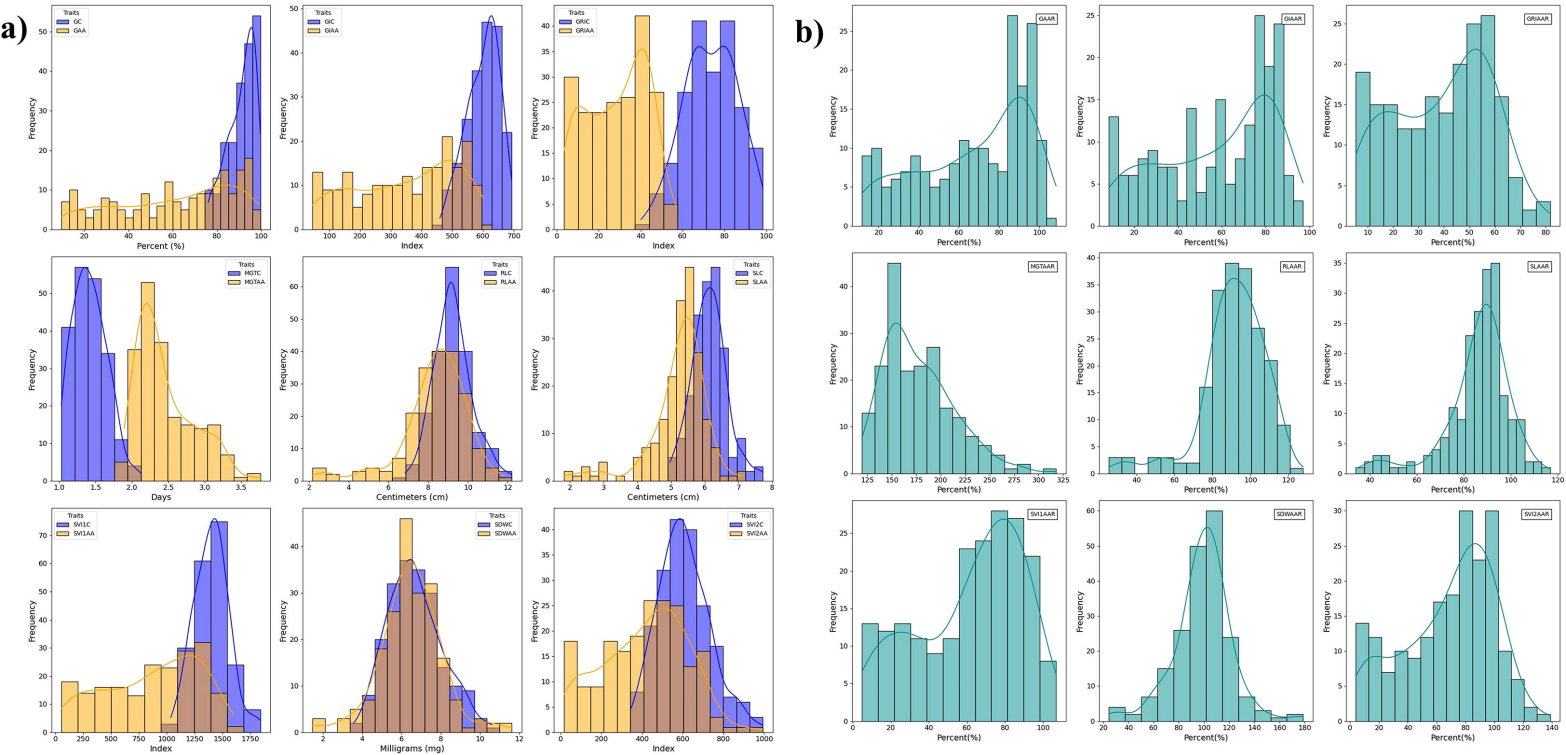
Frequency distribution of seed traits in the GWAS panel. The X-axis represents the trait values and the Y-axis represents the frequency. The traits are organized as follows: **(A)** GC, GAA; GIC, GIAA; GRIC, GRIAA; MGTC, MGTAA; RLC, RLAA; SLC, SLAA; SVI1C, SVI1AA; SDWC, SDWAA; SVI2C, SVI2AA. **(B)** GAAR, GIAAR, GRIAAR, MGTAAR, RLAAR, SLAAR, SVI1AAR, SDWAAR and SVI2AAR.

Phenotypic data distribution of the traits *viz.*, GRIC, RLC, RLAA, SLC, SLAA, SDWC, SDWAA, SVI2C, SLAAR and SDWAAR, closely followed a normal distribution, suggesting the quantitative nature of these traits (**Figure 1**). Descriptive statistics for each trait under control and accelerated aging conditions indicated a consistent trend, with AA negatively impacting seed performance. AA significantly impaired seed performance, decreasing germination percentage and increasing mean germination time. Simultaneously, the root length, shoot length, seedling dry weight and seedling vigor indices exhibited substantial reductions. A few genotypes exhibited resilience to AA treatment, showing minimal reductions in germination percentage, seedling growth and vigor indices. These genotypes maintained stable root and shoot lengths and performed better under AA treatment, demonstrating their potential for use in breeding programs targeting improved seed longevity and stress tolerance (**Figure 1**).

GC ranged from 76.09 to 99.66%, with a mean of 91.07%, indicating good germination potential. GAA exhibited a wider range from 9.65 to 98.89%, with a mean of 61.59%, highlighting the adverse impact of accelerated aging on germination rates. GAAR ranged from 11.67 to 108.40%, with a mean of 67.04%, suggesting that some genotypes maintained high germination rates despite aging (**Supplementary Table S2**). GIC varied from 459.28 to 692.99, with a mean of 599.67. After accelerated aging, the index showed a broader range from 46.14 to 601.56, with a mean of 350.89, reflecting a significant decline due to aging. GIAAR ranged from 8.41 to 97.09%, with a mean of 57.94%. Similarly, GRIC ranged from 40.13 and 98.37, with an average of 73.40. Post-aging GRIAA decreased from 3.39 to 56.89, with a mean of 28.67. GRIAAR ranged from 5.34% to 81.68%, averaging 39.42%. Mean germination time under control ranged from 1.03 to 2.12 days, with a mean of 1.42 days. After accelerated aging it increased to 2.45 days, ranging from 1.89 to 3.76 days. MGTAAR varied from 119.59 to 317.71%, with a mean of 177.67%, highlighting the adverse effects of accelerated aging on germination time.

RLC ranged from 6.84 to 12.21 cm, with a mean of 9.24 cm. After aging, it varied from 2.18 to 11.81 cm, averaging 8.33 cm. RLAAR ranged from 25.37 to 127.72%, with a mean of 91.23%. SLC spanned from 4.97 to 7.73 cm, with a mean of 6.13 cm. After aging, it ranged from 1.82 to 7.08 cm, averaging 5.21 cm. SLAAR with a mean of 85.81% varied between 33.99 and 116.72%. SVI1C ranged from 1035.92 to 1841.18, with a mean of 1400.40. SVI1AA showed a broader range from 53.07 to 1600.15, averaging 873.02, indicating a substantial decline due to aging. SVI1AAR with a mean of 61.62%, spanned from 4.79 to 106.81%. SDWC ranged from 3.56 to 10.60 mg, with a mean of 6.67 mg. SDWAA exhibited a broader range from 1.48 to 11.62 mg, averaging 6.48 mg. SDWAAR ranged from 24.35 to 178.77%, with a mean of 99.20%. SVI2C ranged from 346.65 to 993.87, with a mean of 606.29. After aging, it ranged from 16.47 to 990.25, with an average of 414.95. SVI2AAR varied from 3.95 to 138.62%, averaging 68.76%.

Accelerated aging adversely affected most traits, particularly germination-related parameters, vigor indices and seedling dry weight, as evidenced by the significant reduction in their values post-aging. Analysis of the variance components revealed significant variation among the genotypes for all traits, with high heritability values ranging from 85.09% for SLC to 99.72% for GAAR, indicating a strong genetic influence (**Table 1**).

**Table 1:** Analysis of variance (ANOVA) of 201 pearl millet genotypes for 27 seed phenotypic traits.

Sl. No.	Trait	Sum Sq	Mean Sq	Heritability (%)
1	GC	32494.1	162.5**	96.20
2	GAA	565592.2	2827.9**	99.70
3	GAAR	601723.7	3008.6**	99.72
4	GIC	2359019.0	11795.1**	96.38
5	GIAA	21643691.0	108218.5**	99.62
6	GIAAR	527178.9.0	2635.9**	99.39
7	GRIC	138691.8	693.5**	95.69
8	GRIAA	171866.6	859.3**	99.03
9	GRIAAR	306898.9	1534.5**	98.03
10	MGTC	49.1	0.3**	92.63
11	MGTAA	140.6	0.7**	93.36
12	MGTAAR	1230706.0	6153.5**	91.61
13	RLC	920.2	4.6**	88.39
14	RLAA	2291.1	11.5**	95.20
15	RLAAR	275060.6	1375.3**	91.44
16	SLC	287.0	1.4**	85.09
17	SLAA	653.7	3.3**	94.58
18	SLAAR	191375.0	956.9**	90.15
19	SVI1C	22184860.0	110924.3**	91.60
20	SVI1AA	143998046.0	719990.2**	99.11
21	SVI1AAR	621613.4	3108.1**	98.32
22	SDWC	1528.0	7.6**	98.69
23	SDWAA	1949.4	9.7**	99.36
24	SDWAAR	431261.7	2156.3**	98.08
25	SVI2C	12853410.0	64267.0**	98.15
26	SVI2AA	33841711.0	169208.6**	99.60
27	SVI2AAR	781451.4	3907.3**	99.08

** significance at p = 0.01

**Figure 2:**
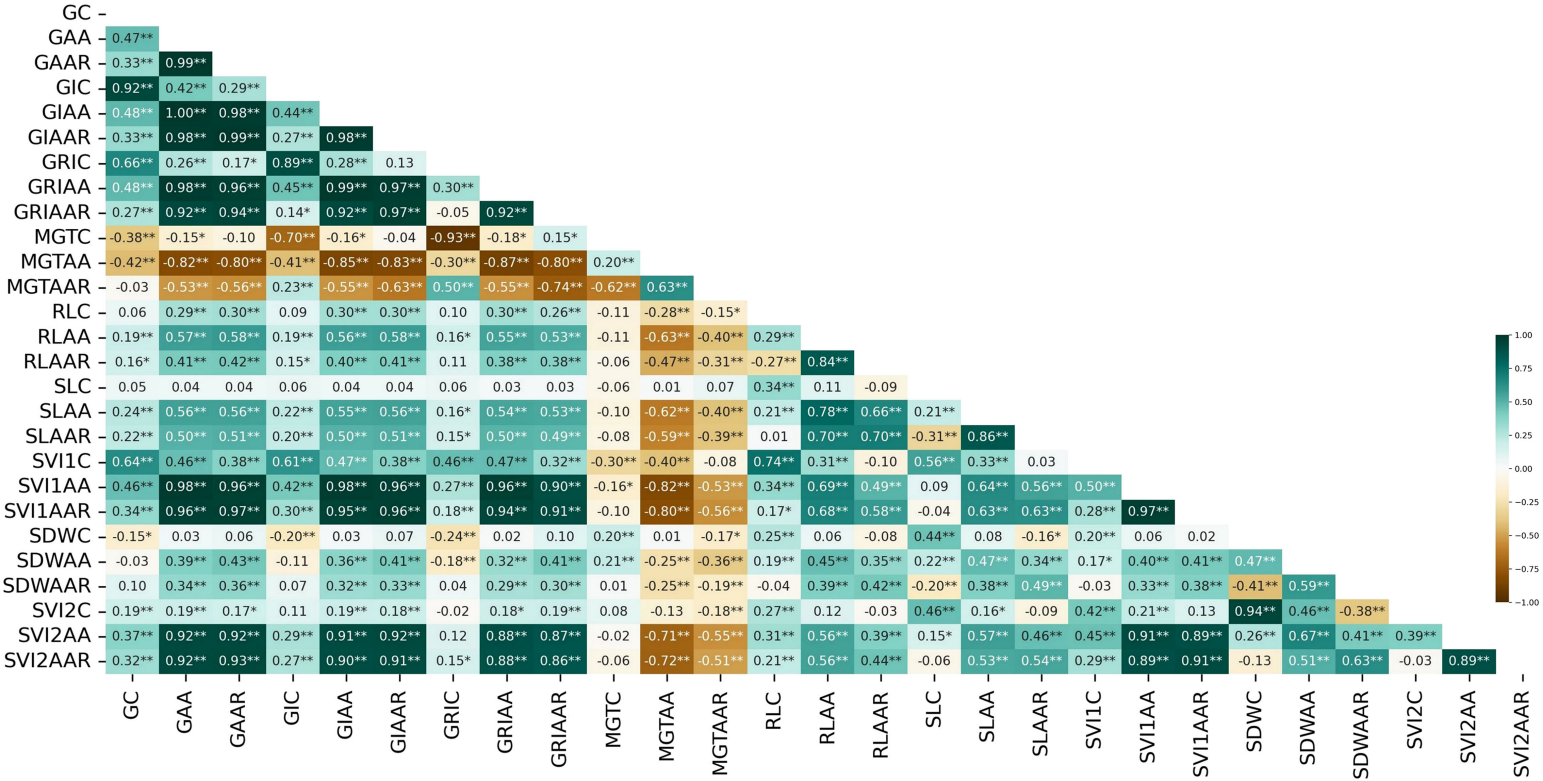
Correlation heatmap representing the relationships between traits, with correlation coefficients ranging from -1 to 1, with darker teal shades representing strong positive correlations and deeper brown shades indicating strong negative correlations. Traits that share high correlation values are marked by stars indicating significance (*p< 0.05, **p< 0.01).

GAA showed significantly positive correlations with SVI2AA (0.92), SVI1AA (0.98), SLAA (0.56) and RLAA (0.57), indicating that these traits are strongly and positively associated and may share similar biological pathways or genetic control (**Figure 2**). In contrast, MGTAA exhibited negative correlations with traits such as GAA (-0.82), GAAR (-0.80), GIAA (-0.85) and GRIAA (-0.55), suggesting an inverse relationship. Additionally, GC showed significant positive correlations with GIC (0.92), GRIC (0.66) and SVI1C (0.64), indicating strong associations among these traits. These correlations offer valuable insights into the intricate relationships between seed traits, which are crucial for optimizing breeding strategies and enhancing crop improvement efforts.

### 3.2 Population structure and genetic relationships

**Figure 3:**
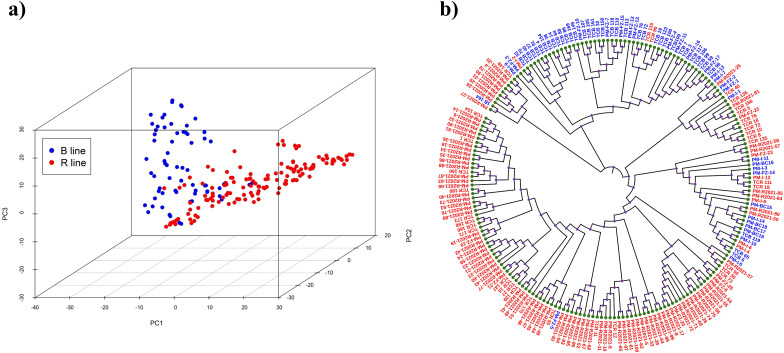
**(A)** Principal components of 201 inbred lines from genotypic data of 2,015 SNPs illustrating the differentiation of B and R lines. **(B)** Neighbor-joining tree of the 201 genotypes showing clustering of B- and R-line genotypes. Branch label colors indicate grouping patterns, with red representing R lines and blue representing B lines.

PCA analysis showed two major groups, represented by the B and R lines and relatively uniform distribution of genotypes, with some overlap between groups (**Figure 3A**). The NJ tree also revealed clustering of B and R-lines in separate groups, indicating the genetic variability between the groups (**Figure 3B**). To account for population stratification, the first 15 PCs and kinship (**Supplementary Figure 1**) values were included as covariates in the MLMM and BLINK models.

### 3.3 Marker trait associations identified through GWAS models

GAPIT model selection feature, which integrates the kinship matrix and PCA, was employed to identify the associations with the traits in a panel of 201 genotypes using 2015 confident SNPs.

**Figure 4:**
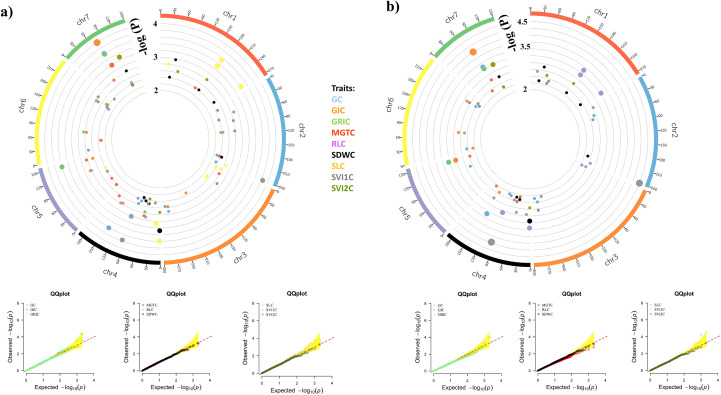
Circos plots showing the distribution of significant MTAs identified under control conditions. Only markers with an adjusted p-value < 0.005 are visualized. The bubble color represents the associated trait, while the bubble size indicates the significance level. Q-Q plots illustrate the deviation between observed and expected p-values for strongly associated markers. **(A)** BLINK model; **(B)** MLMM model.

**Figure 5:**
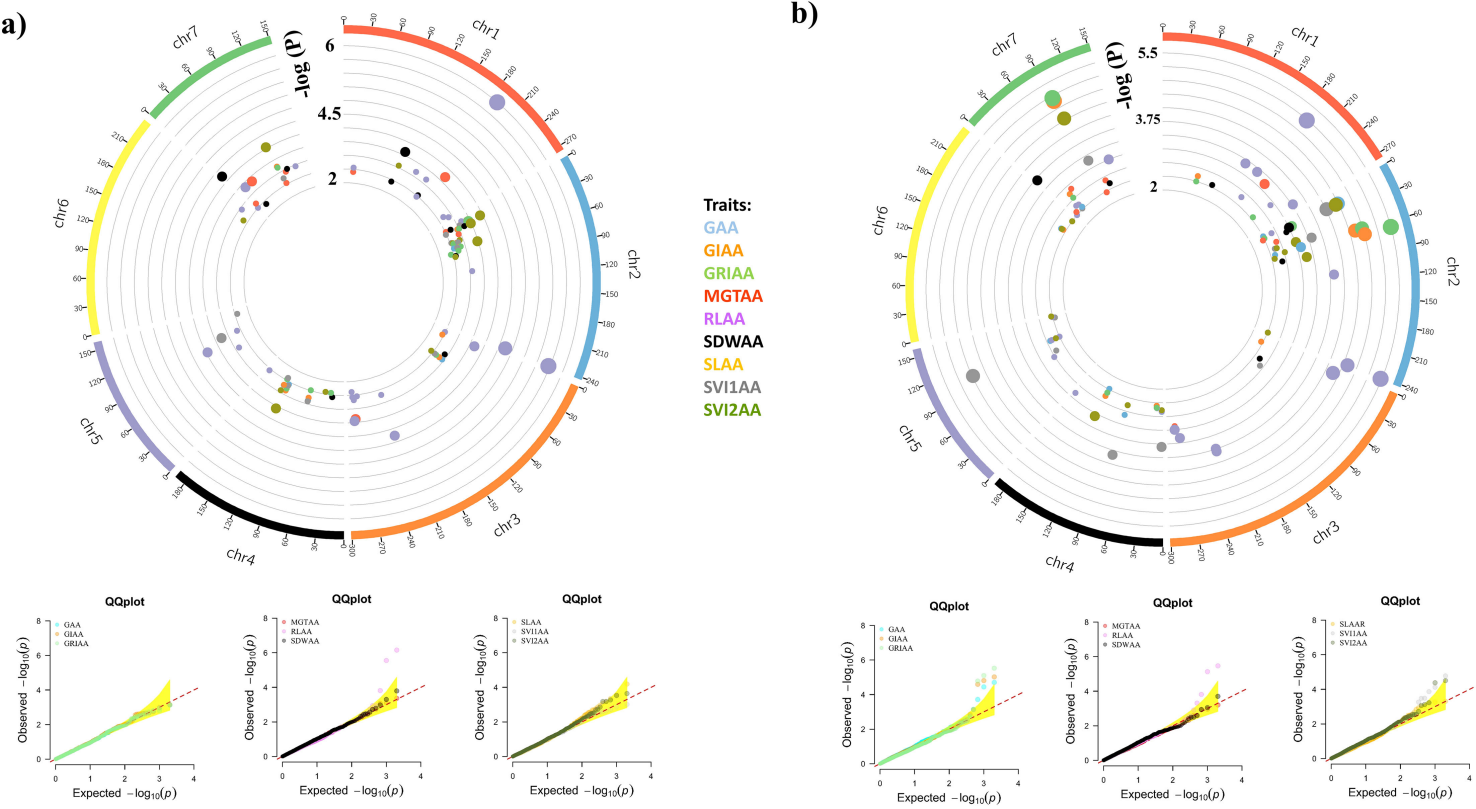
Circos plots showing the distribution of significant MTAs identified under accelerated aging treatment. Only markers with an adjusted p-value < 0.005 are visualized. The bubble color represents the associated trait, while the bubble size indicates the significance level. Q-Q plots illustrate the deviation between observed and expected p-values for strongly associated markers. **(A)** BLINK model; **(B)** MLMM model.

**Figure 6:**
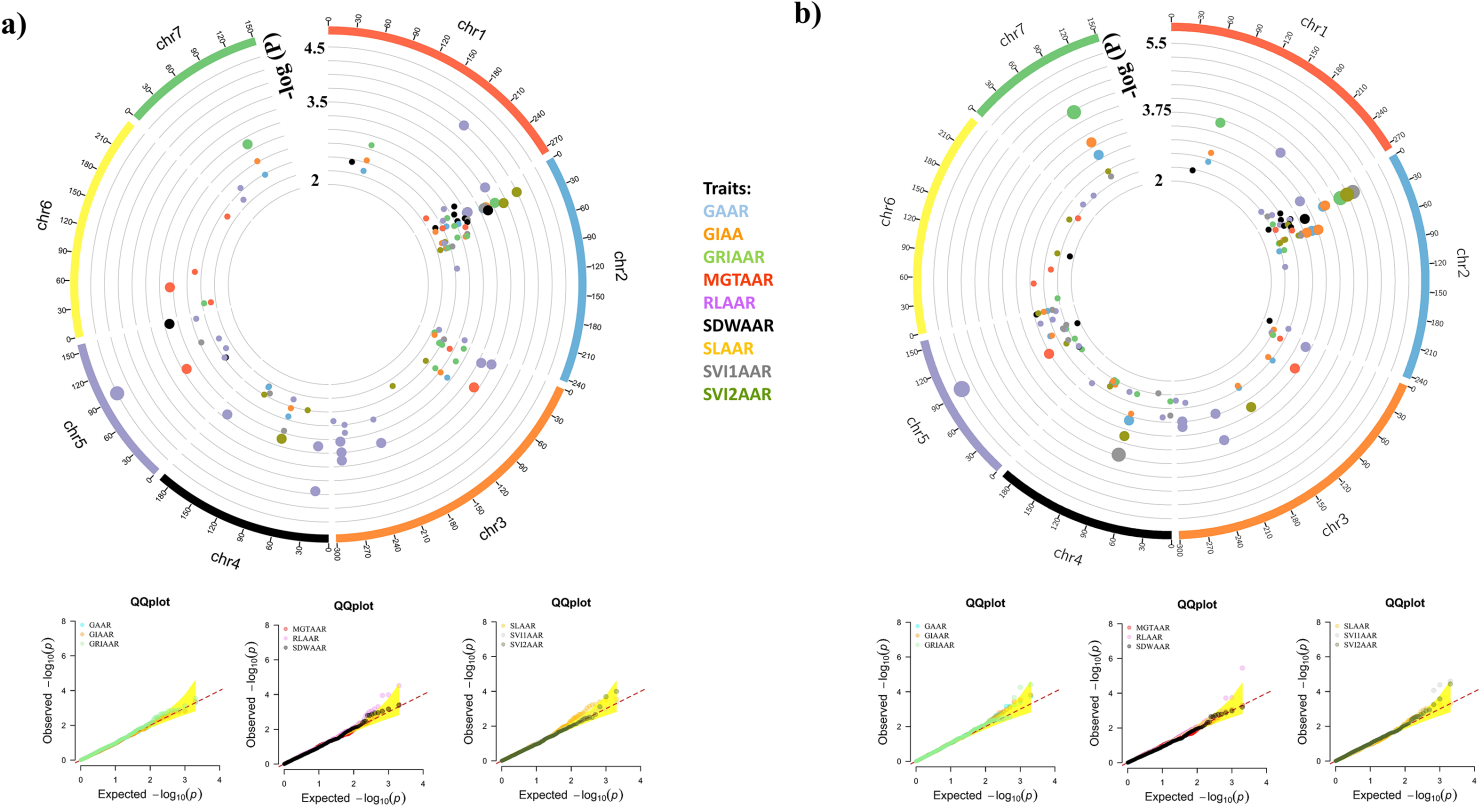
Circos plots showing the distribution of significant MTAs identified for relative measures of accelerated aging treatment. Only markers with an adjusted p-value < 0.005 are visualized. The bubble color represents the associated trait, while the bubble size indicates the significance level. Q-Q plots illustrate the deviation between observed and expected p-values for strongly associated markers. **(A)** BLINK model; **(B)** MLMM model.

A total of 413 significant MTAs were identified for 27 traits using BLINK and MLMM models across seven chromosomes, applying a stringent significance threshold of p< 0.005. Of the 413 significant MTAs identified, 185 were common between the BLINK and MLMM models, while 128 were unique to MLMM and 100 were specific to BLINK. These MTAs were linked to 180 distinct SNPs. GRIAA exhibited the highest number of significant MTAs (32), whereas SVI2C displayed the fewest (4). In addition, chromosome 2 had the highest significant SNPs (38), whereas chromosome 6 had the lowest (15). Detailed MTAs and corresponding p-values are provided in **Supplementary Table S3**. Circos plots depicting the genomic distribution of significant MTAs are presented in **Figures 4**–**6**. Corresponding quantile-quantile (Q–Q) plots illustrate deviations between observed and expected p-values of those MTAs.

PMSnpB394, mapped on chromosome 2 at position 1.58 Mb, is associated with a maximum of 14 traits, namely, GAA, GAAR, GIAAR, GIAA, GRIAA, GRIAAR, MGTAA, MGTAAR, SDWAA, SDWAAR, SVI1AA, SV12AA, SVI1AA and SVI2AAR. Another locus, PMSnpB408 is linked to several traits namely, GAA, GAAR, GIAA, GIAAR, GRIAA, GRIAAR, SDWAA, SDWAAR, SVI1AA, SVI1AAR, SV12AA and SV2AAR. These SNPs exhibit pleiotropic effects, influencing multiple traits simultaneously. PMSnp1505, PMSnpB844 and PMSnp2473 are each associated with 11 traits, including GAA, GAAR, GIAA, SLC and MGTAA. The presence of these pleiotropic SNPs highlights their crucial role in influence on the expression of various seed traits and the shared genetic architecture among these traits. A total of 17 MTAs was identified for GAA, with four common to both the MLMM and BLINK, six unique to BLINK and seven unique to MLMM. For GAAR, 22 MTAs were identified, including five common across models, seven identified by BLINK and 10 by MLMM. Notably, PMSnp2473 and PMSnpB394 on chromosomes 7 and 2 at 8.96 Mb and 15.81 Mb position, respectively, were consistently identified across models for both GAA and GAAR.

For GIAA, 17 MTAs were identified, while GIAAR had 19 SNPs, with eight common between the models. Additionally, 11 SNPs were identified for GIC. PMSnpB394 and PMSnp2473 were significantly associated with GIAA and GIAAR, suggesting their potential role in seed vigor and viability. For MGTC, 11 MTAs were identified, with five common between models and six unique to BLINK. MGTAA was controlled by nine SNPs, with eight common between the models and one unique to BLINK. Similarly, 10 loci were associated with MGTAAR, with eight common between models and two unique to BLINK, indicating their association with mean germination time under accelerated aging stress.

RLAA identified 11 MTAs, seven of which were common to both the BLINK and MLMM models and five identified by BLINK. For RLC, nine SNPs were identified, with six common and one unique to each model. SLAA is associated with 24 loci, with 13 common between models, four unique to BLINK and seven unique to MLMM. PMSnp855 and PMSnpB761 on chromosome 2 and 3 at 242.37 and 9.96 Mb positions, respectively were strongly associated with SLAA, indicating their involvement in seedling growth after accelerated aging stress. Similarly, for SLAAR, 18 MTAs were identified, 16 of which were shared between the two models. PMSnp855 and PMSnpB171 on chromosome 2 and 1, respectively, are associated with both RLAA and SLAA, potentially marking a region of interest for root and seedling length.

A total of 11 MTAs was identified for SDWAA, seven of which were common between models and four were unique to BLINK. For SDWC, 10 SNPs were identified, nine common and one unique to BLINK. PMSnpB2056 on chromosome 7 was consistently identified by the SDWC and SDWAA models. For SVI1C, 20 MTAs were detected, of which three were common between the BLINK and MLMM models, 10 were unique to BLINK and seven were unique to MLMM. SVI1AA revealed 16 SNPs, with seven common, five identified by BLINK and four by MLMM, further illustrating associations with seed vigor. SVI2C had six MTAs, all common between models. SVI2AA identified 23 MTAs, while SVI2AAR revealed 18 SNPs. Notably, significant SNPs (PMSnpB394, PMSnp578, PMSnpB470 and PMSnp563) for SVI2AA were located on chromosome 2, suggesting a key region associated with seed vigor during accelerated aging.

**Figure 7:**
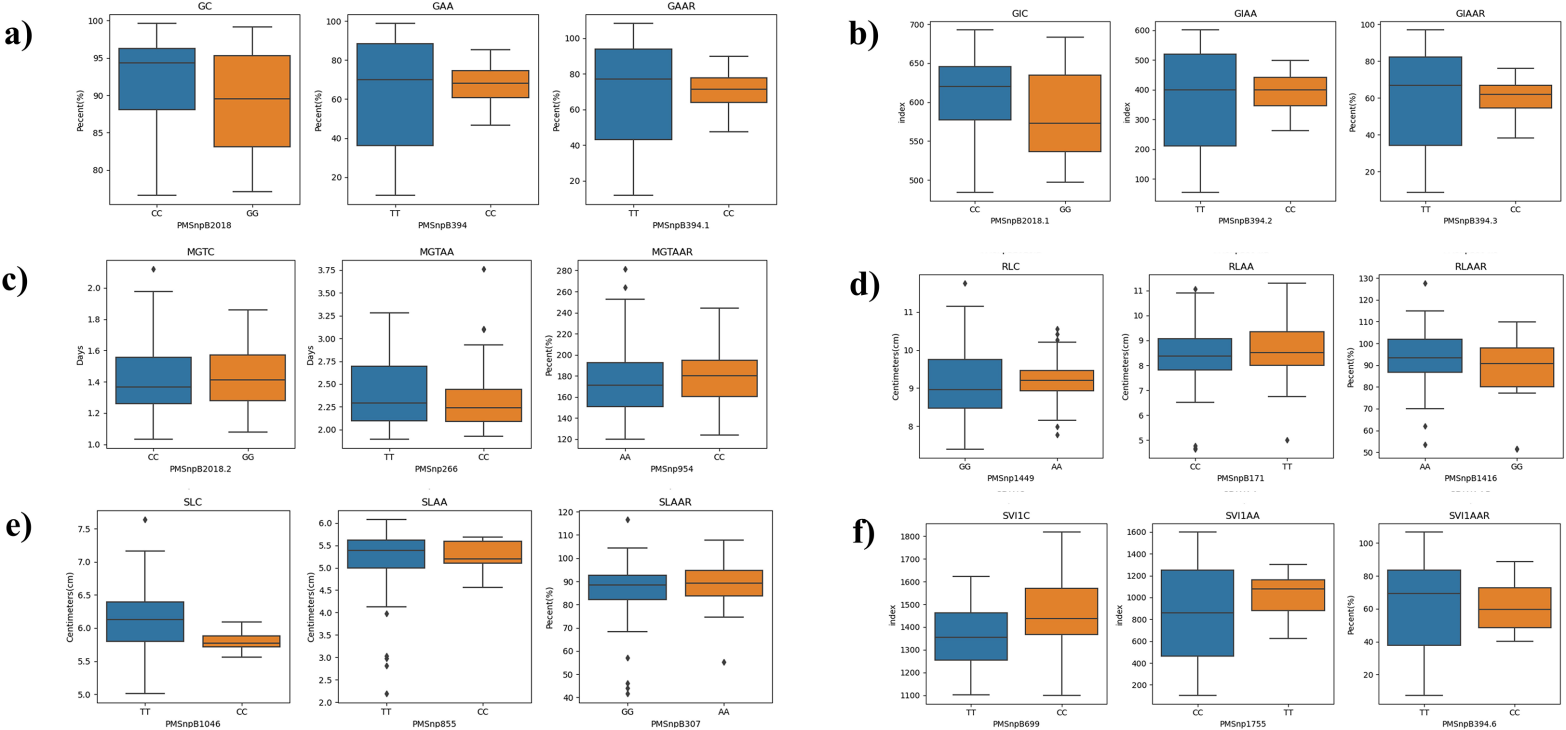
Box plots showing the phenotypic values of the different allele for significant SNPs identified in GWAS for the traits viz., **(A)** GC, GAA, GAAR **(B)** GIC, GIAA, GIAAR, GRIC, GRIAA, GRIAAR **(C)** MGTC, MGTAA, MGTAAR **(D)** RLC, RLAA, RLAAR **(E)** SLC, SLAA, SLAAR, **(F)** SVI1C, SVI1AA, SVI1AAR.The SNP names and alleles are mentioned in the X-axis, with the Y-axis displaying phenotypic values. The black horizontal lines in the middle of the boxes are the median values of the phenotype.

Phenotypic values of different allele classes for the most significant markers in the association panel are examined using boxplots (**Figure 7**). PMSnpB2018 has C and G alleles; genotypes carrying the C allele exhibited higher phenotypic values for GC and GIC, indicating a positive association with the C allele. Similarly, the phenotypic values for PMSnp1755, which has C and T alleles, revealed that genotypes carrying the T allele show significantly higher SVI1AA values, indicating a positive association with the T allele. In contrast, the C allele was associated with reduced phenotypic expression of SVI1AA. These results suggest that PMSnpB2018 and PMSnp1755 could serve as valuable markers for selecting favorable alleles in breeding programs to improve GC, GIC and SVI1AA, respectively.

### 3.4 Candidate gene identification and functional annotation

To identify candidate genes underlying seed longevity and vigor traits, we examined 2Mb upstream and downstream of the genomic regions associated with these traits. Among the 180 significant loci, 124 were localized within predicted gene-coding regions of the reference genome (GCA_002174835.2). The full list of genes located within 2 Mb upstream and downstream of the significant SNPs is available in **Supplementary Table S4**.

**Figure 8:**
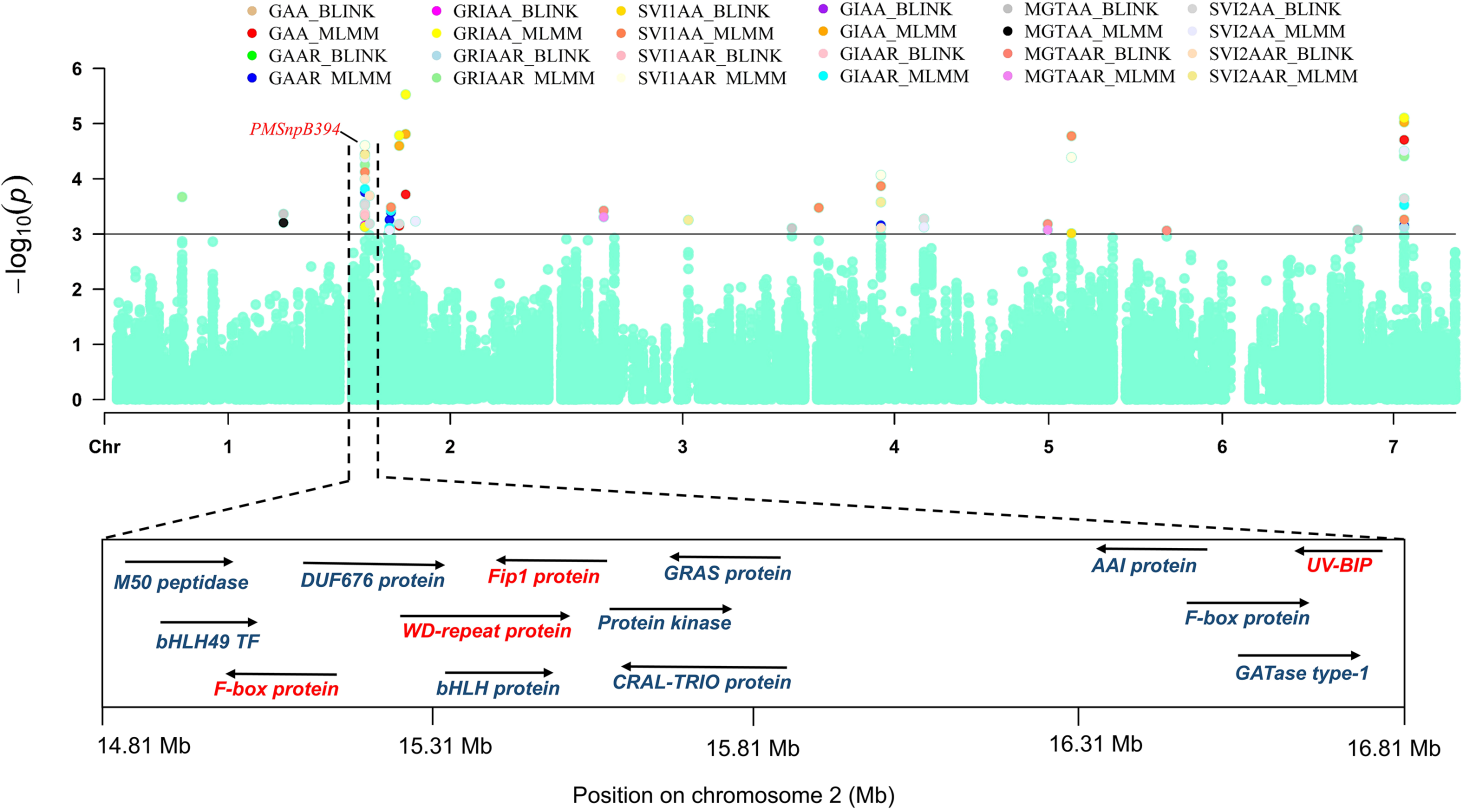
Manhattan plot illustrating the pleiotropic SNP PMSnpB394 on chromosome 2, associated with 14 traits. The genes located within 1 Mb upstream and downstream of the SNP are displayed, with important genes involved in regulating seed longevity and vigour highlighted in red. The arrows indicate the direction of genes on the forward and reverse strands.

One key SNP, PMSnpB394, associated with multiple traits viz., GAA, GAAR, GIAAR, GIAA, GRIAA, GRIAAR, MGTAA, MGTAAR, SDWAA, SDWAAR, SVI1AA, SVI1AAR, SVI2AA and SVI2AAR (**Figure 8**) (**Supplementary Table S5**) is positioned near the candidate genes such as F-box domain-containing protein, WD_REPEATS_REGION domain-containing protein, Fip1 domain-containing protein and UV-B-induced protein. These genes are known to be involved in protein turnover, signal transduction and stress response mechanisms, which collectively contribute to seed longevity.

**Figure 9:**
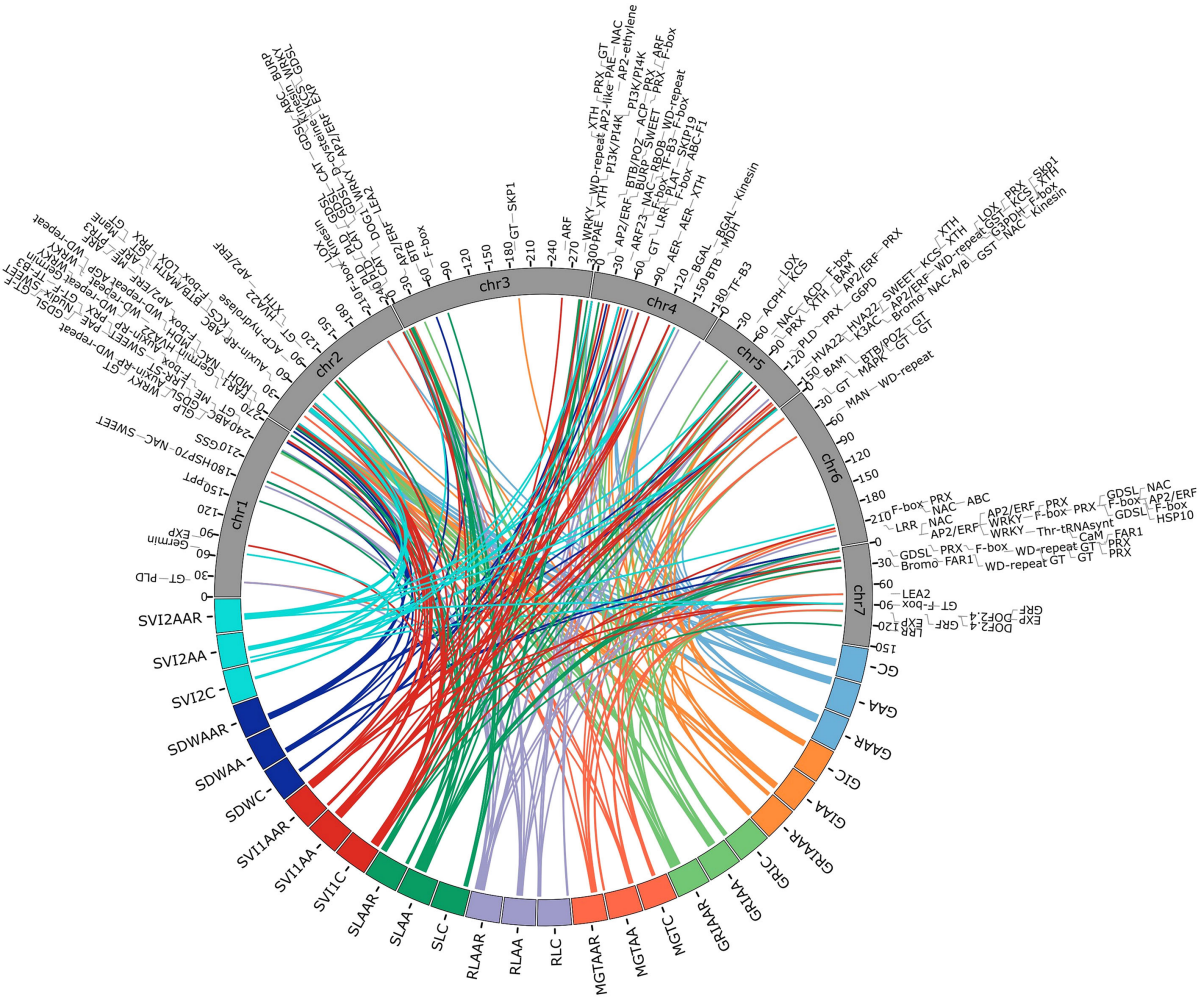
Circos plot visualizing the association of genes on different chromosomes with different seed traits. The lines in the circos plot depict the association between genes on chromosomes with seed traits.

Similarly, PMSnp1505, is closely mapped to the candidate genes, such as Beta-galactosidase and Kinesin motor domain-containing protein, which are implicated in cell wall remodeling and intracellular transport during germination. The pleiotropic SNP PMSnp1435 is linked to an ABC transporter F family member 1, involved in detoxification and lipid transport across membranes, while PMSnp578 is located near Peroxidase and BTB/POZ-MATH domain-containing genes that function in reactive oxygen species (ROS) scavenging and stress adaptation. Together, these associations suggest that genes related to oxidative protection, hormonal signaling and cellular maintenance play crucial roles in maintaining seed vigor and longevity (**Figure 9**; **Supplementary Table S6**).

## 4 Discussion

Seed vigor and longevity are critical factors of crop productivity and play a pivotal role in ensuring sustainable agricultural production. Farmers should have access to quality seeds with high vigor and viability, as seeds carry the genetic potential of any cultivar. Seed vigor and longevity traits are influenced by a complex interplay of genetic, environmental and physiological factors (Reed et al., 2022). Even under optimal storage conditions, seeds will inevitably lose their viability over time due to the processes of seed deterioration or aging (McDonald, 1999). It is estimated that approximately 25% of seeds lose their viability each year, leading to substantial economic losses (McDonald and Nelson, 1986). This emphasizes the importance of investigating the genetic and molecular mechanisms underlying seed aging and vigor to enhance seed longevity, for improving pearl millet productivity especially in the rainfed ecologies.

AA tests are widely employed to simulate long-term storage conditions, enabling the evaluation of seed vigor and longevity (Delouche and Baskin, 1973). Advances in genomics have identified stress-responsive genes, including heat shock proteins, receptor-like kinases and oxidative stress repair mechanisms, that play vital roles in maintaining seed vigor and longevity (Arif et al., 2022). QTLs associated with seed longevity in rice and maize have been shown to influence germination rate, seedling growth and biomass (Liu et al., 2019).

Seed longevity and vigor traits, including GRIC, RLC, RLAA, SLC, SLAA, SDWC, SDWAA, SVI2C, SLAAR and SDWAAR, largely exhibited normal phenotypic distribution pattern. This suggests that these traits are controlled by polygenic inheritance, in which the trait variation arises from the cumulative effects of multiple genes and environmental factors. Traits such as GC, GIC and MGTC displayed skewed distributions, implying that these traits are associated with polygenes exhibiting dominant effects (Allard, 1960).

Accelerated aging consistently reduced seed performance, affecting germination percentage, germination rate indices and seedling vigor traits, indicating the sensitivity of these parameters to storage-induced stress (Rastegar et al., 2011; Waterworth and West, 2023). Germination percentage is a key indicator of a genotype’s potential lifespan following storage-induced aging (Rehmani et al., 2023). The substantial variation observed in germination traits between control and accelerated aging treatments highlights the impact of environmental stresses on seed viability (Landjeva et al., 2010; Zuo et al., 2018; Pirredda et al., 2023). Following aging, the significant reduction in GAA and its relative measure GAAR reflected notable differences in the longevity potential among pearl millet genotypes, which directly influences seed vigor during germination and seedling emergence (Reed et al., 2022). Decline in GIAA and GRIAA, along with their relative measures GIAAR and GRIAAR, reflected weakened seed performance in terms of both germination speed and uniformity critical traits for establishing a uniform crop stand and ensuring optimal productivity (Delouche and Baskin, 1973).

Post-aging decrease in root and shoot lengths emphasized the compromised seedling establishment capacity under stress, which can negatively affect nutrient uptake and overall plant growth (Ivana and Slavoljub, 2012; Ehsan et al., 2017; Chopra et al., 2017). Significant reductions in SVI1AA and SVI2AA further confirm the cumulative impact of oxidative stress on the essential metabolic pathways required for seedling development (Ademola et al., 2021).

Genotypes such as PM-R2021-95, PM-R2021-90, TCR 154 and PM-BC-18 consistently performed well under aging stress, indicating their inherent physiological resilience and superior seed quality (**Table 2**). These top-performing genotypes represent valuable genetic resources for breeding programs aimed at improving seed vigor, germination efficiency and storability in pearl millet, particularly under rainfed and stress-prone environments.

**Table 2:** Superior pearl millet genotypes identified for key traits contributing to seed vigor and longevity.

Sl. No.	Genotype	Traits
1	PM-R2021-95	GIAAR, GRIAAR, SLC, SLAA, SDWC, SDWAA, SVI2C, SVI2AA
2	PM-R2021-90	GIAA, GIAAR, GRIAA, GRIAAR, SVI1AA
3	TCR 154	SLAA, SLAAR, SDWC, SDWAA, SVI2AA
4	PM-R2021-39	RLC, SVI1C, SVI2AAR
5	TCR 156	GC, GIC, GRIC
6	TCR 147	RLAA, SLAA, SLAAR
7	PM-I-9	GC, GIC, RLAA, RLAAR
8	PM-R2021-51	GAA, GAAR, GIAA, SVI1AA
9	PM-BC-18	GAA, GIAA, SVI1AA, SVI1AAR
10	PM-R2021-57	GAAR, GIAAR, GRIAAR

### 4.1 Trait mapping and gene functions

GWAS enable the identification of genetic loci underlying complex quantitative traits by integrating phenotypic variation with genome-wide marker data (Nepolean et al., 2013). In the present study, a coordinated network of genes governing seed vigor, dormancy and longevity was identified. Significant SNPs and their associated genes highlighted diverse molecular pathways involved in hormonal signaling, redox regulation, lipid metabolism and stress-response mechanisms (Kannababu et al., 2025). Several pleiotropic SNPs were identified across for multiple traits, indicating interconnected regulatory networks that collectively sustain seed viability and vigor under stress conditions.

*PMSnpB394* and *PMSnpB490*, on chromosome 2, exhibited pleiotropic effects influencing multiple seed vigor and longevity traits. Genomic region surrounding *PMSnpB394* mapped genes such as F*-box domain-containing proteins*, *WD-repeat region-containing protein*, *Fip1 domain-containing protein* and a *UV-B-induced protein*, which are involved in protein turnover, signal transduction and stress-responsive mechanisms (Finch-Savage and Leubner-Metzger, 2016; Varshney et al., 2023). *PMSnpB490* locus was associated with genes encoding *3-ketoacyl-CoA synthase*, a key enzyme in lipid biosynthesis and seed coat development and a *peptide transporter (PTR3-A*) contributes to nutrient mobilization and seed development (Luo et al., 2024). These pleiotropic loci highlight functionally interconnected genomic regions that regulate seed vigor and longevity traits in pearl millet.

### 4.2 Molecular basis of seed aging and longevity

Regulation of seed aging is governed by a complex network of genes that influence dormancy, germination, stress response and overall seed longevity. These genes can be broadly categorized into various functional groups based on their roles in hormonal signaling, metabolism, transcriptional regulation and structural integrity. During seed aging, a decline in antioxidant enzyme activity (e.g., *catalase, superoxide dismutase* and *peroxidase*) leads to accumulation of reactive oxygen species (ROS), resulting in lipid peroxidation, mitochondrial degradation and inhibition of oxidative phosphorylation (Bailly, 2004; El-Maarouf-Bouteau and Bailly, 2008). This cascade of oxidative damage triggers enzyme inactivation, impairs DNA repair and ultimately reduces seed viability.

**Figure 10:**
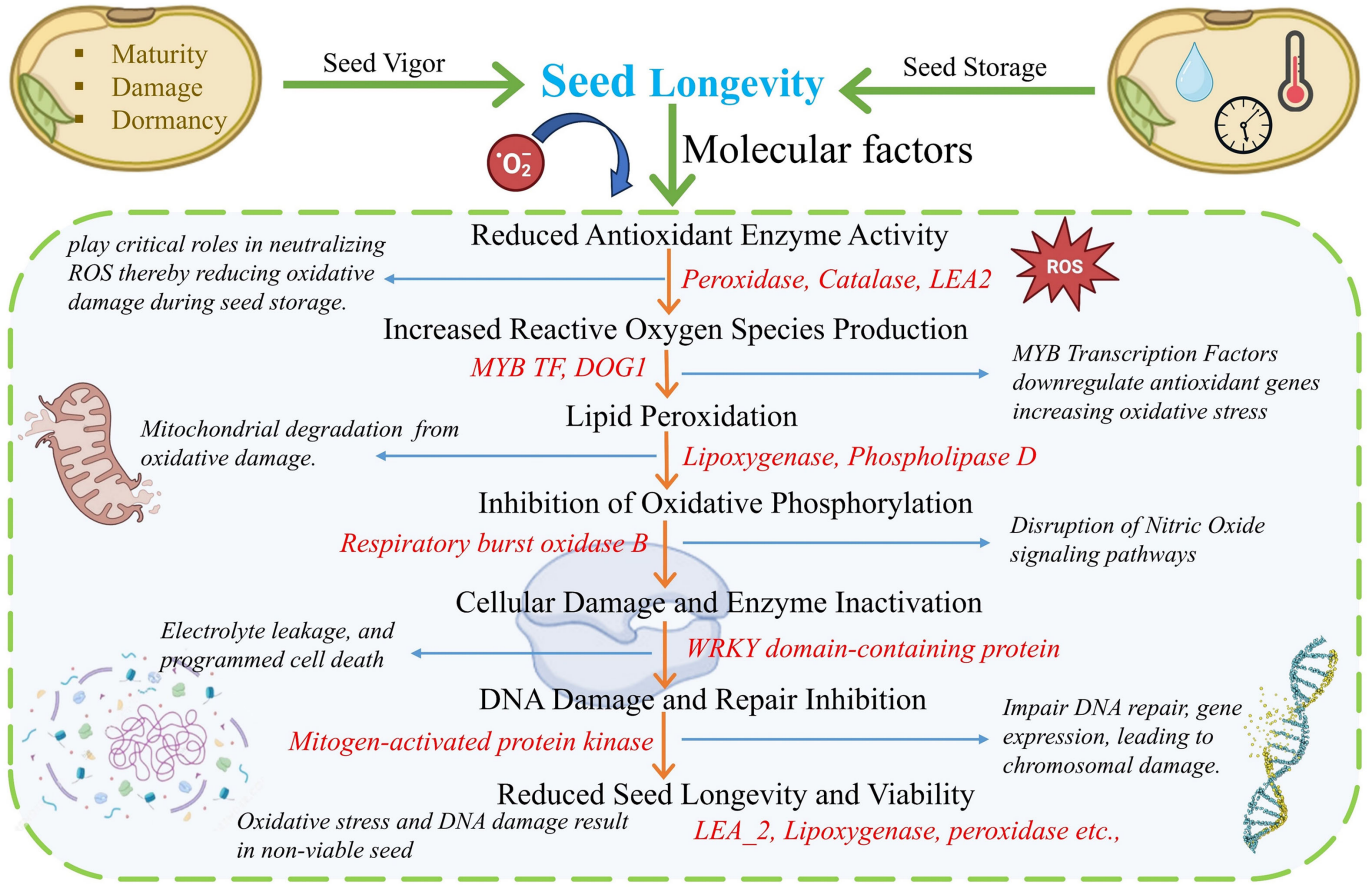
Molecular pathway involved in the regulation of seed longevity, vigor and viability during aging.

Key regulatory factors such as transcription factors *MYB* and *DOG1* are involved in controlling dormancy and germination timing (Nakabayashi et al., 2012), while enzymes such as *lipoxygenase* and *phospholipase D* mediate membrane lipid degradation during oxidative stress (Singh et al., 2022). Similarly, stress-responsive proteins including *WRKY* and *LEA2* help maintain seed vigor and longevity by protecting cellular structures and stabilizing proteins under desiccation and oxidative stress conditions (Huang et al., 2016; Sano et al., 2016). Together, these mechanisms form an integrated defense network that sustains seed viability during prolonged storage (**Figure 10**).

#### 4.2.1 Hormone signaling and regulation

Hormonal pathways play a pivotal role in seed dormancy, germination and stress tolerance. *Pgl_GLEAN_10001638* encoding *Auxin response factor 23*, was mapped by *PMSnpB1166* on chromosome 4, associated with traits SDWC and SVI2C, indicating its role in auxin-mediated regulation of seed vigor. Several genes involved in *auxin* and *abscisic acid* (ABA) signaling pathways have been identified as key regulators. *Auxin response factor* (Liu et al., 2013) and *BTB/POZ* and *MATH domain-containing proteins* (Kim et al., 2015) negatively regulate ABA-mediated inhibition of germination*. Pgl_GLEAN_10006520*, encoding an *HVA22-like protein*, was identified by *PMSnpB1514* and *PMSnpB1515* on chromosome 5, associated with RLAAR, highlighting its potential role in regulating seed dormancy through ABA signaling under abiotic conditions. Other key genes include *DOG1 domain-containing proteins* (Guo and Ho, 2008; Nishimura et al., 2018; Carrillo-Barral et al., 2020) which regulate seed dormancy and germination through ABA signaling, along with *WRKY domain-containing proteins* (Khoso et al., 2022) mediate post-germination growth and stress responses. *AP2/ERF domain-containing proteins* were also identified, highlighting the importance of hormonal crosstalk during germination and root development, consistent with the findings of Ma et al. (2024).

#### 4.2.2 Seed germination and dormancy

A gene cluster was identified on chromosome 5 at 157 Mb regulating seed dormancy, germination and seedling establishment. *Xyloglucan endotransglucosylase/hydrolase* and *GDSL esterase/lipase* promote seed germination and early seedling establishment through the mobilization of stored lipids and endosperm degradation (Zhu et al., 2022; Ding et al., 2023). *PMSnpB1439* mapped *Pgl_GLEAN_10002230*, which encodes a *peroxidase* enzyme, was located at *PMSnpB1439* on chromosome 5 and found to be associated with GC and GIC. This association suggests its involvement in preserving seed viability by facilitating the polymerization of suberin and lignin in the seed coat (Renard et al., 2020b). While, *Glutathione transferase* (Koramutla et al., 2021) mediates redox balance, promoting dormancy release and germination. *Lipoxygenase* (Wang et al., 2023) and *Beta-amylase* (Ali and Elozeiri, 2017) ensure efficient starch degradation, facilitating germination. *Pgl_GLEAN_10030711*, encoding a *lipoxygenase*, mapped on chromosome 5 and exhibited significant associations with GIC, GRIC and MGTC. This gene plays a role in signaling events mediated by lipid peroxidation, which influences seed germination and vigor. This gene contributes to lipid peroxidation–mediated signaling events that modulate seed germination and vigor. Another gene*, Pgl_GLEAN_10037604*, encoding a *β-amylase*, was identified on the same chromosome by *PMSnpB1582*, which is associated with SLAAR and SVI1AA. This finding highlights its crucial role in starch hydrolysis and energy mobilization, essential for early germination processes. Consistent with these findings, *Lipoxygenase* (Wang et al., 2023) and *Beta-amylase* (Ali and Elozeiri, 2017) were reported to ensure efficient starch degradation, thereby facilitating seed germination and vigor.

#### 4.2.3 Stress response and longevity

Seed longevity and stress tolerance are regulated by genes such as *LEA_2 domain-containing proteins*, which are synthesized in response to drought and play a crucial role in maintaining seed viability under adverse conditions (Azarkovich, 2016; Smolikova et al., 2020). *Pgl_GLEAN_10024293*, encoding a *10 kDa heat shock protein*, was closely mapped to *PMSnpB2287* on chromosome 7 and showed an association with MGTAA. This gene protects cellular proteins and preserving seed viability under heat stress conditions. Consistent with this, the *10 kDa* and *70 kDa heat shock proteins* were reported to confer thermotolerance by preventing protein denaturation and ensuring stability during high-temperature conditions (Kaur et al., 2015; Su and Li, 2008). While, *Germin-like proteins* (Wang et al., 2020) regulate dormancy via ABA and gibberellic acid signaling. *Phospholipase D (PLD)* gene plays a key role in lipid degradation during seed aging and its suppression in soybean was improved the long-term seed storage quality (Devaiah et al., 2007; Lee et al., 2012).

#### 4.2.4 Metabolism and seed vigor

Genes involved in seed metabolism are essential for maintaining seed vigor and ensuring robust germination. *Glyceraldehyde-3-phosphate dehydrogenase* (Piattoni et al., 2017) is phosphorylated during seed development, regulating energy metabolism. *Malic enzyme* (Yazdanpanah et al., 2019) and *Glucose-6-phosphate 1-dehydrogenase* (Yang et al., 2019) play critical roles in seed protection against oxidative stress. A pleiotropic locus, *PMSnpB1489* on chromosome 4, was linked to the gene *Pgl_GLEAN_10034303*, encoding a *β-galactosidase* and exhibited significant associations with multiple seed vigor and germination traits, indicating its multifaceted role in regulating early seed development and performance Zhang et al., 2021. This association points to its involvement in cell wall modification and the mobilization of stored carbohydrates, processes that are critical for initiating and sustaining germination. *Pgl_GLEAN_10030031*, encoding an *Expansin*, associated with GRIC and MGTAAC, suggesting its involvement in enhancing germination and seedling growth through cell wall loosening during early developmental stages (Chen and Bradford, 2000). *GDSL domain-containing lipases* (Shen et al., 2022) play a crucial role in coleoptile elongation and root development, thereby contributing significantly to the successful establishment of seedlings.

#### 4.2.5 Transcriptional regulation

Transcription factors play a crucial role in regulating seed traits by modulating gene expression involved in dormancy, germination and early seedling growth. Several transcription factor genes were identified on chromosome 7, including *Pgl_GLEAN_10007407* (*NAC domain-containing protein* associated with SDWAA and SLAA), *Pgl_GLEAN_10007427* (*WRKY domain-containing protein*) and *Pgl_GLEAN_10011162* (*AP2/ERF domain-containing protein*). *TF-B3 domain-containing proteins* (Wei et al., 2023) and *WRKY domain-containing proteins* (Jiang and Yu, 2009) orchestrate various developmental processes, including seed development, germination and dormancy. *NAC domain-containing proteins* (Fuertes-Aguilar and Matilla, 2024) are critical for seed reserve accumulation and dormancy regulation. Furthermore, *Dof zinc finger proteins* (Ruta et al., 2020; Niñoles et al., 2022) are involved in seed development, dormancy and germination, while *GRF-type domain-containing proteins* (Kim et al., 2003) promote cell expansion in cotyledon tissues during early seedling growth.

#### 4.2.6 Seed coat and structural integrity

Structural components of seeds play a crucial role in determining their longevity, dormancy, and stress resilience*. Pgl_GLEAN_10030730*, encoding a 3*-ketoacyl-CoA synthase*, associated with GIC, GRIC and MGTC, indicating its involvement in maintaining seed coat integrity and enforcing physical dormancy. This gene was identified by PMSnpB1556 on chromosome 5. *Pgl_GLEAN_10002158*, encoding a *bidirectional sugar transporter SWEET*, was mapped within genomic regions associated with seed vigor traits, had a role in carbohydrate mobilization and energy balance during germination. Supporting these findings, *Peroxidase* (Naghisharifi et al., 2023) contributes to seed coat integrity by catalyzing the polymerization of suberin, polyphenolics and lignin, while *3-ketoacyl-CoA synthase* (Chai et al., 2021) is known to maintain seed coat structure and physical dormancy. *Pectin acetylesterase* (Palmer et al., 2021) modulates seed coat permeability, influencing water uptake and dormancy release. *F-box domain-containing proteins* (Gong et al., 2022) inhibit germination and seedling growth under stress, highlighting their role in maintaining seed structural integrity during environmental challenges.

## 5 Conclusions

Seed vigor and longevity are crucial factors affecting pearl millet crop establishment, especially in rainfed ecologies, and play a vital role in the sustainable production of grain and fodder. Substantial phenotypic variation was observed among 201 genotypes, with heritability estimates ranging from 85.09% (SLC) to 99.72% (GAAR). Genome-wide association analysis uncovered 413 significant SNP–trait associations, of which 185 were consistently detected across both BLINK and MLMM models, indicating a complex polygenic architecture. The identification of the pleiotropic SNP *PMSnpB394*, associated with 14 vigor and longevity-related traits, highlights genomic regions of major effect influencing seed performance and viability.

The study revealed the involvement of key genes such as *lipoxygenase*, *3-ketoacyl-CoA synthase*, *β-galactosidase* and *10 kDa heat shock protein*, which are implicated in stress tolerance, lipid metabolism, and dormancy regulation. These genes collectively outline the molecular networks that safeguard seed and promote successful germination under prolonged storage. Collectively, these Our findings provide molecular targets for enhancing seed vigor and longevity through marker-assisted selection and gene-editing strategies, thereby improving crop resilience and ensuring sustainable pearl millet production under variable environmental conditions.

## Data Availability

The datasets presented in this study can be found in online repositories. The names of the repository/repositories and accession number(s) can be found in the article/**Supplementary Material**.
