# Supplementary material for: Pleiotropic SNPs and genes linked to enhanced seed longevity and vigor for climate resilient pearl millet production
Source: Front Plant Sci. 2026 Mar 31;17:1717105. doi: 10.3389/fpls.2026.1717105 (PMC13076119; doi:10.3389/fpls.2026.1717105)
Supplement: Supplementary file 1 [file DataSheet1.pdf]

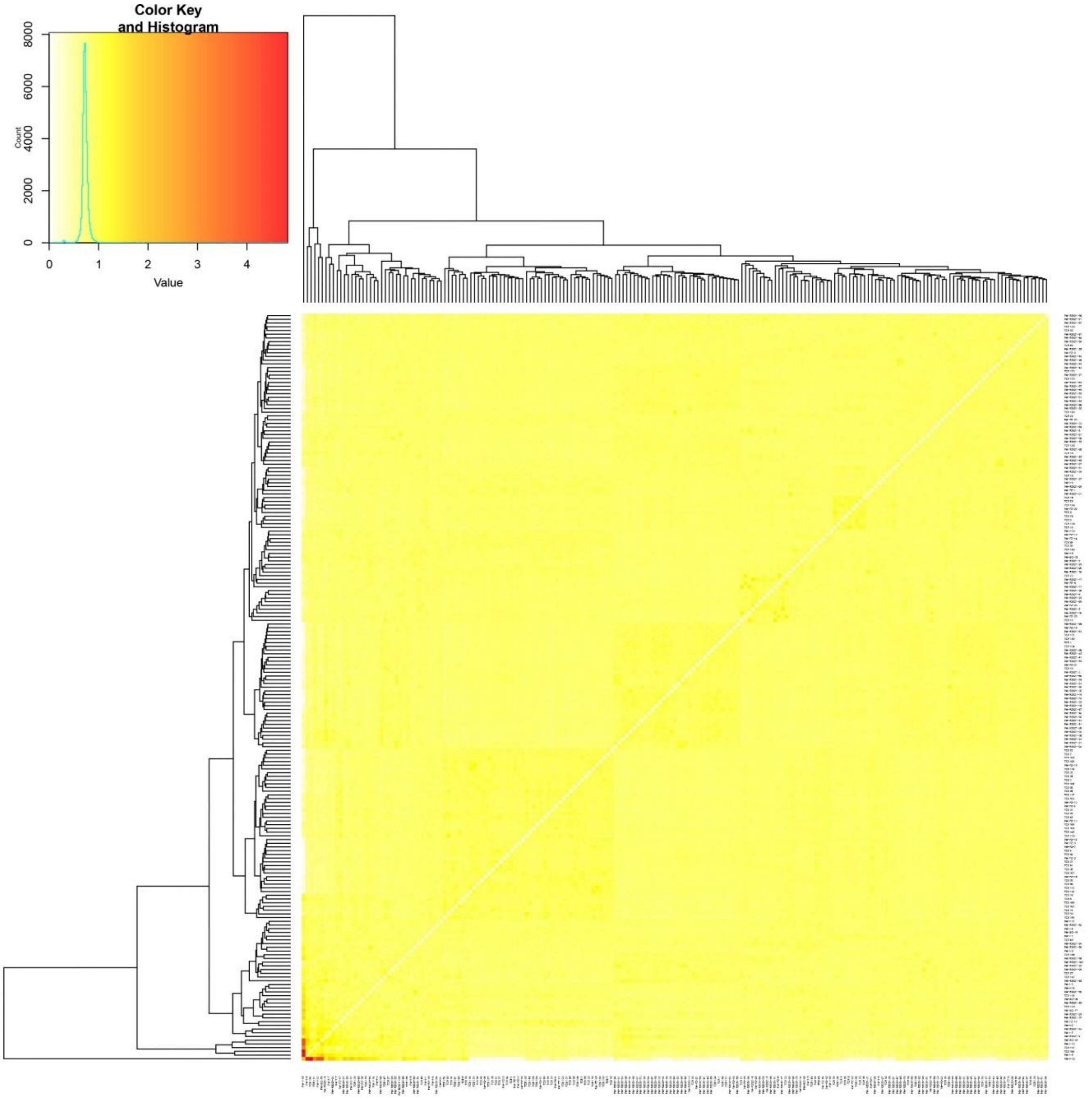

Supplementary Figure 1: Kinship matrix of 201 genotypes constructed from 2,015 SNPs, illustrating overall low genetic relatedness among genotypes, as indicated by cooler colors

Supplementary Table S1: Comprehensive list of B and R lines from the pearl millet GWAS panel.

Sl. No.	Inbred	Group	Sl. No.	Inbred	Group	Sl. No.	Inbred	Group	Sl. No.	Inbred	Group
**1**	TCB 3	B line	**51**	TCR 46	R line	**101**	PM-R2021-14	R line	**151**	PM-R2021-67	R line
**2**	TCB 4	B line	**52**	TCR 50	R line	**102**	PM-R2021-17	R line	**152**	PM-R2021-68	R line
**3**	TCB 6	B line	**53**	TCR 55	R line	**103**	PM-R2021-18	R line	**153**	PM-R2021-69	R line
**4**	TCB 7	B line	**54**	TCR 72	R line	**104**	PM-R2021-19	R line	**154**	PM-R2021-71	R line
**5**	TCB 14	B line	**55**	TCR 73	R line	**105**	PM-R2021-20	R line	**155**	PM-R2021-72	R line
**6**	TCB 19	B line	**56**	TCR 76	R line	**106**	PM-R2021-21	R line	**156**	PM-R2021-73	R line
**7**	TCB 32	B line	**57**	TCR 90	R line	**107**	PM-R2021-22	R line	**157**	PM-R2021-74	R line
**8**	TCB 33	B line	**58**	TCR 111	R line	**108**	PM-R2021-23	R line	**158**	PM-R2021-75	R line
**9**	TCB 37	B line	**59**	TCR 115	R line	**109**	PM-R2021-24	R line	**159**	PM-R2021-76	R line
**10**	TCB 38	B line	**60**	TCR 135	R line	**110**	PM-R2021-25	R line	**160**	PM-R2021-77	R line
**11**	TCB 45	B line	**61**	TCR 136	R line	**111**	PM-R2021-26	R line	**161**	PM-R2021-79	R line
**12**	TCB 59	B line	**62**	TCR 139	R line	**112**	PM-R2021-27	R line	**162**	PM-R2021-80	R line
**13**	TCB 65	B line	**63**	TCR 147	R line	**113**	PM-R2021-28	R line	**163**	PM-R2021-81	R line
**14**	TCB 70	B line	**64**	TCR 149	R line	**114**	PM-R2021-29	R line	**164**	PM-R2021-84	R line
**15**	TCB 72	B line	**65**	TCR 150	R line	**115**	PM-R2021-30	R line	**165**	PM-R2021-85	R line
**16**	TCB 76	B line	**66**	TCR 154	R line	**116**	PM-R2021-31	R line	**166**	PM-R2021-86	R line
**17**	TCB 84	B line	**67**	TCR 156	R line	**117**	PM-R2021-32	R line	**167**	PM-R2021-87	R line
**18**	TCB 86	B line	**68**	TCR 158	R line	**118**	PM-R2021-33	R line	**168**	PM-R2021-88	R line
**19**	TCB 88	B line	**69**	TCR 166	R line	**119**	PM-R2021-34	R line	**169**	PM-R2021-89	R line
**20**	TCB 91	B line	**70**	TCR 170	R line	**120**	PM-R2021-35	R line	**170**	PM-R2021-90	R line
**21**	TCB 96	B line	**71**	TCR 171	R line	**121**	PM-R2021-36	R line	**171**	PM-R2021-91	R line
**22**	TCB 99	B line	**72**	TCR 173	R line	**122**	PM-R2021-37	R line	**172**	PM-R2021-92	R line
**23**	TCB 100	B line	**73**	TCR 180	R line	**123**	PM-R2021-39	R line	**173**	PM-R2021-93	R line
**24**	TCB 105	B line	**74**	PM-I-1	B line	**124**	PM-R2021-40	R line	**174**	PM-R2021-94	R line
**25**	TCB 106	B line	**75**	PM-I-2	R line	**125**	PM-R2021-41	R line	**175**	PM-R2021-95	R line
**26**	TCB 107	B line	**76**	PM-I-3	B line	**126**	PM-R2021-42	R line	**176**	PM-R2021-96	R line
**27**	TCB 109	B line	**77**	PM-I-4	R line	**127**	PM-R2021-43	R line	**177**	PM-R2021-97	R line
**28**	TCB 110	B line	**78**	PM-I-5	B line	**128**	PM-R2021-44	R line	**178**	PM-R2021-98	R line
**29**	TCB 113	B line	**79**	PM-I-6	R line	**129**	PM-R2021-45	R line	**179**	PM-R2021-99	R line
**30**	TCB 117	B line	**80**	PM-I-7	B line	**130**	PM-R2021-46	R line	**180**	PM-R2021-100	R line
**31**	TCB 118	B line	**81**	PM-I-8	R line	**131**	PM-R2021-47	R line	**181**	PM-FZ-1	B line
**32**	TCB 119	B line	**82**	PM-I-9	B line	**132**	PM-R2021-48	R line	**182**	PM-FZ-2	B line
**33**	TCB 123	B line	**83**	PM-I-10	B line	**133**	PM-R2021-49	R line	**183**	PM-FZ-3	B line
**34**	TCB 149	B line	**84**	PM-I-11	B line	**134**	PM-R2021-50	R line	**184**	PM-FZ-4	B line
**35**	TCB 150	B line	**85**	PM-I-12	R line	**135**	PM-R2021-51	R line	**185**	PM-FZ-5	B line
**36**	TCB 154	B line	**86**	PM-I-13	B line	**136**	PM-R2021-52	R line	**186**	PM-FZ-6	R line
**37**	TCB 161	B line	**87**	PM-I-14	B line	**137**	PM-R2021-53	R line	**187**	PM-FZ-7	B line
**38**	TCB 164	B line	**88**	PM-BC-15	B line	**138**	PM-R2021-54	R line	**188**	PM-FZ-10	B line
**39**	TCB 169	B line	**89**	PM-BC-16	B line	**139**	PM-R2021-55	R line	**189**	PM-FZ-11	B line
**40**	TCR 1	R line	**90**	PM-BC-17	B line	**140**	PM-R2021-56	R line	**190**	PM-FZ-12	B line
**41**	TCR 5	R line	**91**	PM-BC-18	B line	**141**	PM-R2021-57	R line	**191**	PM-FZ-13	B line
**42**	TCR 8	R line	**92**	PM-BC-19	B line	**142**	PM-R2021-58	R line	**192**	PM-FZ-14	B line

Supplementary Table S1. Continued….

**43**	TCR 10	R line	**93**	PM-R2021-3	R line	**143**	PM-R2021-59	R line	**193**	PM-FZ-15	B line
**44**	TCR 11	R line	**94**	PM-R2021-4	R line	**144**	PM-R2021-60	R line	**194**	PM-FZ-16	B line
**45**	TCR 12	R line	**95**	PM-R2021-5	R line	**145**	PM-R2021-61	R line	**195**	PM-FZ-17	B line
**46**	TCR 15	R line	**96**	PM-R2021-6	R line	**146**	PM-R2021-62	R line	**196**	PM-FZ-19	R line
**47**	TCR 16	R line	**97**	PM-R2021-8	R line	**147**	PM-R2021-63	R line	**197**	PM-FZ-20	R line
**48**	TCR 18	R line	**98**	PM-R2021-9	R line	**148**	PM-R2021-64	R line	**198**	PM-FZ-21	R line
**49**	TCR 27	R line	**99**	PM-R2021-10	R line	**149**	PM-R2021-65	R line	**199**	PM-FZ-22	R line
**50**	TCR 30	R line	**100**	PM-R2021-11	R line	**150**	PM-R2021-66	R line	**200**	PM-FZ-23	R line
									**201**	PM-FZ-25	R line

Supplementary Table S2: Summary of the descriptive statistics for phenotypic traits measured across 201 pearl millet accessions from the GWAS panel.

Sl. No.	Trait	Min	Max	Range	Mean	SD
1	GC	76.09	99.66	23.57	91.07	6.12
2	GAA	9.65	98.89	89.24	61.59	26.45
3	GAAR	11.67	108.4	96.73	67.04	27.23
4	GIC	459.28	692.99	233.72	599.67	52.21
5	GIAA	46.14	601.56	555.42	350.89	163.46
6	GIAAR	8.41	97.09	88.69	57.94	25.44
7	GRIC	40.13	98.37	58.25	73.4	12.57
8	GRIAA	3.39	56.89	53.5	28.67	14.48
9	GRIAAR	5.34	81.68	76.33	39.42	19.15
10	MGTC	1.03	2.12	1.09	1.42	0.23
11	MGTAA	1.89	3.76	1.87	2.45	0.39
12	MGTAAR	119.59	317.71	198.12	177.67	35.85
13	RLC	6.84	12.21	5.36	9.24	0.95
14	RLAA	2.18	11.81	9.63	8.33	1.61
15	RLAAR	25.37	127.72	102.35	91.23	16.91
16	SLC	4.97	7.73	2.75	6.13	0.51
17	SLAA	1.82	7.08	5.25	5.21	0.85
18	SLAAR	33.99	116.72	82.73	85.81	13.91
19	SVI1C	1035.92	1841.18	805.26	1400.4	152.16
20	SVI1AA	53.07	1600.15	1547.08	873.02	419.43
21	SVI1AAR	4.79	106.81	102.02	61.62	27.34
22	SDWC	3.56	10.6	7.04	6.67	1.36
23	SDWAA	1.48	11.62	10.14	6.48	1.55
24	SDWAAR	24.35	178.77	154.43	99.2	22.72
25	SVI2C	346.65	993.87	647.22	606.29	124.11
26	SVI2AA	16.47	990.25	973.78	414.95	204.34
27	SVI2AAR	3.95	138.62	134.68	68.76	30.89

Min = Minimum: Max= Maximum: SD= Standard deviation

Supplementary Table S3: Detailed List of Significant MTAs along with p-values Identified Using MLMM and BLINK models.

Sl. No.	Trait	SNP ID	Model	Chr	Position	P value	-log P value
**1**	**GAA**	PMSnp2473	BLINK	7	89618943	0.00156	2.807
			MLMM	7	89618943	0.00002	4.706
		PMSnpB394	BLINK	2	15812931	0.000744	3.128
			MLMM	2	15812931	0.000035	4.44
		PMSnp1435	BLINK	4	79201633	0.002045	2.689
			MLMM	4	79201633	0.001586	2.8
		PMSnp578	BLINK	2	58312350	0.003448	2.462
			MLMM	2	58312350	0.000713	3.147
		PMSnpB408	BLINK	2	21500078	0.001786	2.748
		PMSnpB844	BLINK	3	65928346	0.001812	2.742
		PMSnpB2111	BLINK	7	152636920	0.002149	2.668
		PMSnpB490	BLINK	2	48142589	0.002621	2.582
		PMSnp1505	BLINK	4	141674641	0.002754	2.56
		PMSnp1489	BLINK	4	132541695	0.003351	2.475
		PMSnpB532	MLMM	2	58312350	0.003448	2.462
		PMSnp1755	MLMM	5	107579664	0.002442	2.612
		PMSnpB1047	MLMM	4	2360127	0.002616	2.582
		PMSnp2169	MLMM	6	201271649	0.003301	2.481
		PMSnp2330	MLMM	7	22779127	0.003941	2.404
		PMSnpB358	MLMM	2	7652856	0.004374	2.359
		PMSnp1489	MLMM	4	132541695	0.004637	2.334
**2**	**GAAR**	PMSnpB394	BLINK	2	15812931	0.000474	3.324
			MLMM	2	15812931	0.000176	3.754
		PMSnpB490	BLINK	2	48142589	0.001461	2.835
			MLMM	2	48142589	0.00041	3.387
		PMSnp2473	BLINK	7	89618943	0.003275	2.485
			MLMM	7	89618943	0.000698	3.156
		PMSnpB470	BLINK	2	44592445	0.003745	2.427
			MLMM	2	44592445	0.003014	2.521
		PMSnp1505	BLINK	4	1.42E+08	0.004536	2.343
			MLMM	4	141674641	0.003469	2.46
		PMSnpB844	BLINK	3	65928346	0.001181	2.928
		PMSnpB408	BLINK	2	21500078	0.00162	2.791
		PMSnp1435	BLINK	4	79201633	0.002046	2.689
		PMSnp486	BLINK	2	15631126	0.002667	2.574
		PMSnpB2111	BLINK	7	1.53E+08	0.003116	2.506
		PMSnp578	BLINK	2	58312350	0.00383	2.417
		PMSnpB113	BLINK	1	80656275	0.004531	2.344
		PMSnp563	MLMM	2	46269689	0.000555	3.256
		PMSnp1435	MLMM	4	79201633	0.000692	3.16
		PMSnp1755	MLMM	5	107579664	0.001443	2.841
		PMSnpB844	MLMM	3	65928346	0.001857	2.731

		PMSnpB1611	MLMM	5	158614523	0.002039	2.691
		PMSnpB863	MLMM	3	157525175	0.002138	2.67
		PMSnpB113	MLMM	1	80656275	0.00236	2.627
		PMSnpB470	MLMM	2	44592445	0.003014	2.521
		PMSnpB1247	MLMM	4	133408245	0.004277	2.369
		PMSnpB532	MLMM	2	66322133	0.004953	2.305
**3**	**GC**	PMSnpB2018	BLINK	7	74229314	0.000243	3.614
			MLMM	7	74229314	0.000444	3.353
		PMSnp1505	BLINK	4	141674641	0.000245	3.611
			MLMM	4	141674641	0.000448	3.348
		PMSnpB462	BLINK	2	42668341	0.001306	2.884
			MLMM	2	42668341	0.002117	2.674
		PMSnpB1439	BLINK	5	108567735	0.001561	2.807
			MLMM	5	108567735	0.002554	2.593
		PMSnp1943	BLINK	6	15340226	0.001904	2.72
			MLMM	6	15340226	0.00289	2.539
		PMSnpB955	BLINK	3	278704801	0.001908	2.72
			MLMM	3	278704801	0.003161	2.5
		PMSnp2169	BLINK	6	201271649	0.001939	2.712
			MLMM	6	201271649	0.003074	2.512
		PMSnpB1219	BLINK	4	87512075	0.002647	2.577
			MLMM	4	87512075	0.004244	2.372
		PMSnpB990	BLINK	3	289909985	0.002746	2.561
			MLMM	3	289909985	0.00432	2.365
		PMSnpB1214	BLINK	4	78940729	0.003357	2.474
		PMSnpB586	BLINK	2	108551940	0.003742	2.427
		PMSnp2189	BLINK	6	210323969	0.003754	2.426
		PMSnp854	BLINK	2	242048635	0.004563	2.341
		PMSnp781	BLINK	2	211741286	0.004963	2.304
**4**	**GIAA**	PMSnpB394	BLINK	2	15812931	0.000717	3.145
			MLMM	2	15812931	0.000747	3.127
		PMSnp2473	BLINK	7	89618943	0.001034	2.985
			MLMM	7	89618943	0.000009	5.02
		PMSnp1489	BLINK	4	132541695	0.002118	2.674
			MLMM	4	132541695	0.00288	2.541
		PMSnp578	BLINK	2	58312350	0.002415	2.617
			MLMM	2	58312350	0.000025	4.6
		PMSnpB408	BLINK	2	21500078	0.001762	2.754
		PMSnpB2111	BLINK	7	152636920	0.002225	2.653
		PMSnpB844	BLINK	3	65928346	0.002293	2.64
		PMSnp1435	BLINK	4	79201633	0.002451	2.611
		PMSnpB490	BLINK	2	48142589	0.002532	2.597
		PMSnp1505	BLINK	4	141674641	0.002781	2.556
		PMSnpB784	BLINK	3	18489047	0.004394	2.357
		PMSnpB532	MLMM	2	66322133	0.000016	4.81
		PMSnp2330	MLMM	7	22779127	0.001383	2.859

		PMSnpB113	MLMM	1	80656275	0.003236	2.49
		PMSnpB1076	MLMM	4	13968214	0.003722	2.429
		PMSnp2169	MLMM	6	201271649	0.003816	2.418
		PMSnpB782	MLMM	3	18165957	0.004978	2.303
**5**	**GIAAR**	PMSnpB394	BLINK	2	15812931	0.000428	3.369
			MLMM	2	15812931	0.000153	3.814
		PMSnpB490	BLINK	2	48142589	0.001501	2.824
			MLMM	2	48142589	0.000382	3.418
		PMSnpB844	BLINK	3	65928346	0.001688	2.773
			MLMM	3	65928346	0.002577	2.589
		PMSnp2473	BLINK	7	89618943	0.001705	2.768
			MLMM	7	89618943	0.000298	3.525
		PMSnpB113	BLINK	1	80656275	0.002867	2.543
			MLMM	1	80656275	0.001361	2.866
		PMSnp1435	BLINK	4	79201633	0.002946	2.531
			MLMM	4	79201633	0.00104	2.983
		PMSnpB470	BLINK	2	44592445	0.004183	2.379
			MLMM	2	44592445	0.003227	2.491
		PMSnpB750	BLINK	3	6572955	0.004801	2.319
			MLMM	3	6572955	0.004553	2.342
		PMSnpB408	BLINK	2	21500078	0.001974	2.705
		PMSnpB2111	BLINK	7	152636920	0.003268	2.486
		PMSnp578	BLINK	2	58312350	0.003642	2.439
		PMSnp486	BLINK	2	15631126	0.004448	2.352
		PMSnp563	MLMM	2	46269689	0.000762	3.118
		PMSnp1755	MLMM	5	107579664	0.001603	2.795
		PMSnpB1611	MLMM	5	158614523	0.001616	2.792
		PMSnpB863	MLMM	3	157525175	0.002436	2.613
		PMSnpB1247	MLMM	4	133408245	0.003539	2.451
		PMSnp1505	MLMM	4	141674641	0.003942	2.404
		PMSnpB784	MLMM	3	18489047	0.004313	2.365
**6**	**GIC**	PMSnpB2018	BLINK	7	74229314	0.000047	4.328
			MLMM	7	74229314	0.000097	4.01
		PMSnp2169	BLINK	6	201271649	0.00165	2.782
			MLMM	6	201271649	0.001272	2.895
		PMSnp1505	BLINK	4	141674641	0.001731	2.762
			MLMM	4	141674641	0.003673	2.435
		PMSnpB1753	BLINK	6	81117759	0.001739	2.76
			MLMM	6	81117759	0.001474	2.831
		PMSnpB1556	BLINK	5	148528570	0.002245	2.649
			MLMM	5	148528570	0.000944	3.025
		PMSnpB1439	BLINK	5	108567735	0.00143	2.845
		PMSnpB955	BLINK	3	278704801	0.002654	2.576
		PMSnp2189	BLINK	6	210323969	0.00344	2.463
		PMSnpB586	BLINK	2	108551940	0.004364	2.36
		PMSnpB871	BLINK	3	189492464	0.00471	2.327

		PMSnp1941	MLMM	6	14665488	0.004846	2.315
**7**	**GRIAA**	PMSnpB394	BLINK	2	15812931	0.000701	3.154
			MLMM	2	15812931	0.000737	3.132
		PMSnp2473	BLINK	7	89618943	0.001111	2.954
			MLMM	7	89618943	0.000008	5.1
		PMSnp578	BLINK	2	58312350	0.002165	2.665
			MLMM	2	58312350	0.000016	4.78
		PMSnp1489	BLINK	4	132541695	0.002462	2.609
			MLMM	4	132541695	0.003581	2.446
		PMSnpB532	BLINK	2	66322133	0.002701	2.568
			MLMM	2	66322133	0.000003	5.53
		PMSnpB408	BLINK	2	21500078	0.001557	2.808
		PMSnpB844	BLINK	3	65928346	0.003023	2.52
		PMSnpB490	BLINK	2	48142589	0.003257	2.487
		PMSnpB2111	BLINK	7	152636920	0.003445	2.463
		PMSnp1435	BLINK	4	79201633	0.004102	2.387
		PMSnp1505	BLINK	4	141674641	0.004325	2.364
		PMSnp1340	BLINK	4	30943673	0.004543	2.343
		PMSnpB541	BLINK	2	70809535	0.004699	2.328
		PMSnp2330	MLMM	7	22779127	0.001863	2.73
		PMSnpB1076	MLMM	4	13968214	0.003316	2.479
		PMSnp317	MLMM	1	240246150	0.003886	2.41
		PMSnp477	MLMM	2	13222807	0.004349	2.362
		PMSnpB113	MLMM	1	80656275	0.004496	2.347
**8**	**GRIAAR**	PMSnpB394	BLINK	2	15812931	0.00028	3.552
			MLMM	2	15812931	0.000056	4.25
		PMSnp2473	BLINK	7	89618943	0.000754	3.123
			MLMM	7	89618943	0.000039	4.41
		PMSnpB490	BLINK	2	48142589	0.001398	2.855
			MLMM	2	48142589	0.004407	2.356
		PMSnpB113	BLINK	1	80656275	0.001473	2.832
			MLMM	1	80656275	0.000213	3.671
		PMSnpB470	BLINK	2	44592445	0.0021	2.678
			MLMM	2	44592445	0.002889	2.539
		PMSnpB784	BLINK	3	18489047	0.002992	2.524
			MLMM	3	18489047	0.004108	2.386
		PMSnpB1649	BLINK	6	15555727	0.003416	2.466
			MLMM	6	15555727	0.004145	2.382
		PMSnpB750	BLINK	3	6572955	0.001243	2.906
		PMSnpB799	BLINK	3	33237448	0.001295	2.888
		PMSnpB408	BLINK	2	21500078	0.00195	2.71
		PMSnp436	BLINK	2	1562601	0.002203	2.657
		PMSnpB844	BLINK	3	65928346	0.002229	2.652
		PMSnp578	BLINK	2	58312350	0.003465	2.46
		PMSnp910	BLINK	3	18137908	0.00347	2.46
		PMSnpB2111	BLINK	7	152636920	0.004087	2.389

		PMSnpB731	BLINK	3	1230694	0.004994	2.302
		PMSnpB1047	MLMM	4	2360127	0.003029	2.519
		PMSnp1435	MLMM	4	79201633	0.003464	2.46
		PMSnpB532	MLMM	2	66322133	0.003687	2.433
		PMSnpB337	MLMM	2	1568337	0.003856	2.414
		PMSnp1755	MLMM	5	107579664	0.004061	2.391
		PMSnpB1247	MLMM	4	133408245	0.004237	2.373
		PMSnp1498	MLMM	4	139901663	0.004373	2.359
		PMSnp1631	MLMM	5	47175943	0.004821	2.317
		PMSnpB1363	MLMM	5	78147411	0.004839	2.315
**9**	**GRIC**	PMSnpB1556	BLINK	5	148528570	0.000251	3.6
			MLMM	5	148528570	0.000511	3.292
		PMSnpB2018	BLINK	7	74229314	0.00026	3.586
			MLMM	7	74229314	0.000526	3.279
		PMSnpB1753	BLINK	6	81117759	0.001421	2.847
			MLMM	6	81117759	0.002489	2.604
		PMSnpB1186	BLINK	4	56564602	0.001713	2.766
			MLMM	4	56564602	0.002952	2.53
		PMSnp1584	BLINK	5	7396868	0.003603	2.443
		PMSnpB2049	BLINK	7	101505710	0.004132	2.384
		PMSnp2002	BLINK	6	51038162	0.004892	2.311
**10**	**MGTAA**	PMSnp266	BLINK	1	206077539	0.000435	3.361
			MLMM	1	206077539	0.000626	3.203
		PMSnpB969	BLINK	3	285669819	0.000787	3.104
			MLMM	3	285669819	0.0011	2.959
		PMSnp2358	BLINK	7	31936267	0.000843	3.074
			MLMM	7	31936267	0.001176	2.93
		PMSnpB436	BLINK	2	31806717	0.001851	2.733
			MLMM	2	31806717	0.002487	2.604
		PMSnpB2046	BLINK	7	100625770	0.001851	2.733
			MLMM	7	100625770	0.002488	2.604
		PMSnp2287	BLINK	7	4892399	0.002926	2.534
			MLMM	7	4892399	0.003847	2.415
		PMSnp2473	BLINK	7	89618943	0.003644	2.438
			MLMM	7	89618943	0.004743	2.324
		PMSnpB394	BLINK	2	15812931	0.003786	2.422
			MLMM	2	15812931	0.004919	2.308
		PMSnpB40	BLINK	1	23107173	0.004721	2.326
**11**	**MGTAAR**	PMSnp954	BLINK	3	52727204	0.000378	3.423
			MLMM	3	52727204	0.000494	3.306
		PMSnpB1363	BLINK	5	78147411	0.000661	3.18
			MLMM	5	78147411	0.000848	3.072
		PMSnp2002	BLINK	6	51038162	0.000867	3.062
			MLMM	6	51038162	0.001102	2.958
		PMSnpB436	BLINK	2	31806717	0.001309	2.883
			MLMM	2	31806717	0.001641	2.785

		PMSnpB784	BLINK	3	18489047	0.002094	2.679
			MLMM	3	18489047	0.002583	2.588
		PMSnpB1753	BLINK	6	81117759	0.002409	2.618
			MLMM	6	81117759	0.002957	2.529
		PMSnpB394	BLINK	2	15812931	0.003166	2.5
			MLMM	2	15812931	0.003851	2.414
		PMSnp2200	BLINK	6	214735171	0.004035	2.394
			MLMM	6	214735171	0.004869	2.313
		PMSnpB1648	BLINK	6	15538744	0.004561	2.341
		PMSnpB264	BLINK	1	261869507	0.004715	2.327
**12**	**MGTC**	PMSnpB2018	BLINK	7	74229314	0.000641	3.193
			MLMM	7	74229314	0.001629	2.788
		PMSnpB1186	BLINK	4	56564602	0.001305	2.885
			MLMM	4	56564602	0.00303	2.519
		PMSnpB1556	BLINK	5	148528570	0.001437	2.842
			MLMM	5	148528570	0.003297	2.482
		PMSnpB2005	BLINK	7	51159742	0.001608	2.794
			MLMM	7	51159742	0.003636	2.439
		PMSnpB1267	BLINK	4	168384453	0.002302	2.638
			MLMM	4	168384453	0.004976	2.303
		PMSnp929	BLINK	3	30314876	0.002606	2.584
		PMSnp1584	BLINK	5	7396868	0.00374	2.427
		PMSnpB1322	BLINK	5	40450908	0.003909	2.408
		PMSnpB145	BLINK	1	159919849	0.00395	2.403
		PMSnp790	BLINK	2	212897884	0.004157	2.381
		PMSnp2002	BLINK	6	51038162	0.004685	2.329
**13**	**RLAA**	PMSnpB171	BLINK	1	189340908	0.000001	6.158
			MLMM	1	189340908	0.000007	5.13
		PMSnp855	BLINK	2	242376490	0.000003	5.552
			MLMM	2	242376490	0.000003	5.47
		PMSnpB902	BLINK	3	222710962	0.000154	3.812
			MLMM	3	222710962	0.000154	3.812
		PMSnpB163	BLINK	1	181457906	0.001149	2.94
			MLMM	1	181457906	0.000491	3.309
		PMSnp1755	BLINK	5	107579664	0.003098	2.509
			MLMM	5	107579664	0.002729	2.564
		PMSnpB1295	BLINK	4	188189158	0.003514	2.454
			MLMM	4	188189158	0.001231	2.91
		PMSnpB137	BLINK	1	155857614	0.001131	2.947
		PMSnp2224	BLINK	6	222426222	0.001809	2.743
		PMSnpB40	BLINK	1	23107173	0.003516	2.454
		PMSnpB761	BLINK	3	9960062	0.004104	2.387
		PMSnpB988	BLINK	3	289497585	0.00498	2.303
**14**	**RLAAR**	PMSnpB1416	BLINK	5	94721841	0.000031	4.512
			MLMM	5	94721841	0.000004	5.45
		PMSnpB171	BLINK	1	189340908	0.000105	3.981

			MLMM	1	189340908	0.000182	3.741
		PMSnpB1276	BLINK	4	177549602	0.00067	3.174
			MLMM	4	177549602	0.001801	2.744
		PMSnpB902	BLINK	3	222710962	0.001699	2.77
			MLMM	3	222710962	0.00019	3.721
		PMSnp437	BLINK	2	2086409	0.002714	2.566
			MLMM	2	2086409	0.002265	2.645
		PMSnp1317	BLINK	4	17219968	0.000113	3.947
		PMSnp892	BLINK	3	14887346	0.000495	3.306
		PMSnpB979	BLINK	3	288497651	0.000897	3.047
		PMSnpB374	BLINK	2	9853626	0.000961	3.017
		PMSnpB956	BLINK	3	279191384	0.001273	2.895
		PMSnp1674	BLINK	5	72938324	0.004246	2.372
		PMSnp1435	BLINK	4	79201633	0.004321	2.364
		PMSnp2322	BLINK	7	18966344	0.004344	2.362
		PMSnp855	BLINK	2	242376490	0.004392	2.357
		PMSnpB1515	MLMM	5	137862688	0.001109	2.955
		PMSnpB1514	MLMM	5	137856230	0.002362	2.627
		PMSnpB374	MLMM	2	9853626	0.002713	2.566
		PMSnp1449	MLMM	4	97274250	0.003377	2.471
		PMSnpB1441	MLMM	5	108735664	0.003559	2.449
		PMSnpB1885	MLMM	7	115383	0.003782	2.422
		PMSnpB227	MLMM	1	245488590	0.004539	2.343
**15**	**RLC**	PMSnp1449	BLINK	4	97274250	0.000974	3.011
			MLMM	4	97274250	0.000874	3.059
		PMSnpB1172	BLINK	4	50614031	0.00122	2.914
			MLMM	4	50614031	0.001222	2.913
		PMSnpB1259	BLINK	4	149156871	0.00163	2.788
			MLMM	4	149156871	0.001918	2.717
		PMSnpB2051	BLINK	7	103354573	0.003558	2.449
			MLMM	7	103354573	0.00451	2.346
		PMSnpB1231	BLINK	4	97269148	0.003663	2.436
			MLMM	4	97269148	0.004745	2.324
		PMSnp1416	BLINK	4	68828420	0.004073	2.39
			MLMM	4	68828420	0.003269	2.486
		PMSnp2268	BLINK	7	964079	0.00426	2.371
		PMSnpB1799	BLINK	6	196523288	0.004617	2.336
		PMSnp472	MLMM	2	12660776	0.001702	2.769
**16**	**SDWAA**	PMSnp2266	BLINK	7	555912	0.000161	3.792
			MLMM	7	555912	0.000198	3.702
		PMSnp155	BLINK	1	118546611	0.000501	3.3
			MLMM	1	118546611	0.003895	2.41
		PMSnpB408	BLINK	2	21500078	0.001049	2.979
			MLMM	2	21500078	0.001149	2.94
		PMSnpB2056	BLINK	7	105879125	0.00162	2.791
			MLMM	7	105879125	0.003236	2.49

		PMSnp959	BLINK	3	53664387	0.001915	2.718
			MLMM	3	53664387	0.00316	2.5
		PMSnpB394	BLINK	2	15812931	0.002692	2.57
			MLMM	2	15812931	0.000895	3.048
		PMSnp620	BLINK	2	76274721	0.003485	2.458
			MLMM	2	76274721	0.002545	2.594
		PMSnpB1113	BLINK	4	26907845	0.003434	2.464
		PMSnpB171	BLINK	1	189340908	0.0037	2.432
		PMSnpB122	BLINK	1	118545657	0.004518	2.345
		PMSnp2325	BLINK	7	20593751	0.004957	2.305
**17**	**SDWAAR**	PMSnpB408	BLINK	2	21500078	0.000414	3.383
			MLMM	2	21500078	0.00063	3.201
		PMSnpB1608	BLINK	5	157082717	0.000696	3.157
			MLMM	5	157082717	0.00103	2.987
		PMSnpB371	BLINK	2	9697453	0.000926	3.033
			MLMM	2	9697453	0.001349	2.87
		PMSnpB420	BLINK	2	24937725	0.001117	2.952
			MLMM	2	24937725	0.00161	2.793
		PMSnpB395	BLINK	2	17150912	0.001154	2.938
			MLMM	2	17150912	0.001662	2.779
		PMSnpB307	BLINK	1	272635807	0.001336	2.874
			MLMM	1	272635807	0.001909	2.719
		PMSnp437	BLINK	2	2086409	0.00159	2.799
			MLMM	2	2086409	0.002249	2.648
		PMSnpB394	BLINK	2	15812931	0.001598	2.796
			MLMM	2	15812931	0.00226	2.646
		PMSnp1653	BLINK	5	56333264	0.003405	2.468
			MLMM	5	56333264	0.004623	2.335
		PMSnp90	BLINK	1	51133649	0.00353	2.452
			MLMM	1	51133649	0.004785	2.32
		PMSnpB360	BLINK	2	7965411	0.004162	2.381
			MLMM	2	7965411	0.005591	2.253
		PMSnp850	MLMM	2	241026638	0.007015	2.154
		PMSnpB1776	MLMM	6	119751831	0.007771	2.11
		PMSnpB1441	MLMM	5	108735664	0.008428	2.074
**18**	**SDWC**	PMSnpB1046	BLINK	4	2335729	0.000523	3.281
			MLMM	4	2335729	0.000651	3.187
		PMSnp90	BLINK	1	51133649	0.001077	2.968
			MLMM	1	51133649	0.001311	2.882
		PMSnpB2056	BLINK	7	105879125	0.001483	2.829
			MLMM	7	105879125	0.001789	2.747
		PMSnpB1166	BLINK	4	46294843	0.003136	2.504
			MLMM	4	46294843	0.003702	2.432
		PMSnp357	BLINK	1	259662433	0.003234	2.49
			MLMM	1	259662433	0.003814	2.419
		PMSnpB648	BLINK	2	214885103	0.00351	2.455

			MLMM	2	214885103	0.00413	2.384
		PMSnpB1191	BLINK	4	61588322	0.00364	2.439
			MLMM	4	61588322	0.004279	2.369
		PMSnpB60	BLINK	1	39471473	0.003785	2.422
			MLMM	1	39471473	0.004444	2.352
		PMSnp225	BLINK	1	177680520	0.003815	2.418
			MLMM	1	177680520	0.004479	2.349
		PMSnpB1207	BLINK	4	73301103	0.004429	2.354
**19**	**SLAA**	PMSnp855	BLINK	2	242376490	0.000065	4.189
			MLMM	2	242376490	0.000027	4.56
		PMSnpB761	BLINK	3	9960062	0.000454	3.343
			MLMM	3	9960062	0.00005	4.3
		PMSnpB1952	BLINK	7	14950256	0.000823	3.084
			MLMM	7	14950256	0.002942	2.531
		PMSnpB374	BLINK	2	9853626	0.0011	2.959
			MLMM	2	9853626	0.002592	2.586
		PMSnpB594	BLINK	2	114680996	0.001383	2.859
			MLMM	2	114680996	0.000142	3.849
		PMSnpB2077	BLINK	7	124413159	0.001729	2.762
			MLMM	7	124413159	0.000877	3.057
		PMSnp1674	BLINK	5	72938324	0.002038	2.691
			MLMM	5	72938324	0.001924	2.716
		PMSnpB274	BLINK	1	266066748	0.002682	2.572
			MLMM	1	266066748	0.000371	3.431
		PMSnpB902	BLINK	3	222710962	0.002755	2.56
			MLMM	3	222710962	0.00018	3.744
		PMSnpB971	BLINK	3	286154027	0.002863	2.543
			MLMM	3	286154027	0.000889	3.051
		PMSnpB953	BLINK	3	278207076	0.003619	2.441
			MLMM	3	278207076	0.000529	3.276
		PMSnp2266	BLINK	7	555912	0.003933	2.405
			MLMM	7	555912	0.004423	2.354
		PMSnpB171	BLINK	1	189340908	0.004216	2.375
			MLMM	1	189340908	0.002772	2.557
		PMSnpB1416	BLINK	5	94721841	0.000276	3.559
		PMSnpB977	BLINK	3	288052364	0.000696	3.157
		PMSnpB307	BLINK	1	272635807	0.002017	2.695
		PMSnpB979	BLINK	3	288497651	0.003469	2.46
		PMSnpB137	MLMM	1	155857614	0.000489	3.31
		PMSnpB224	MLMM	1	243224170	0.001109	2.955
		PMSnpB1047	MLMM	4	2360127	0.002656	2.576
		PMSnp2325	MLMM	7	20593751	0.003119	2.506
		PMSnpB1073	MLMM	4	12913262	0.003243	2.489
		PMSnpB1606	MLMM	5	157024018	0.004332	2.363
		PMSnp1755	MLMM	5	107579664	0.004458	2.351
**20**	**SLAAR**	PMSnpB307	BLINK	1	272635807	0.000293	3.533

			MLMM	1	272635807	0.000492	3.308
		PMSnpB761	BLINK	3	9960062	0.000328	3.484
			MLMM	3	9960062	0.000547	3.262
		PMSnpB979	BLINK	3	288497651	0.000405	3.392
			MLMM	3	288497651	0.000667	3.176
		PMSnpB977	BLINK	3	288052364	0.000571	3.243
			MLMM	3	288052364	0.000919	3.037
		PMSnpB902	BLINK	3	222710962	0.000604	3.219
			MLMM	3	222710962	0.000969	3.014
		PMSnp1317	BLINK	4	17219968	0.000743	3.129
			MLMM	4	17219968	0.001176	2.93
		PMSnpB374	BLINK	2	9853626	0.000928	3.033
			MLMM	2	9853626	0.001448	2.839
		PMSnp855	BLINK	2	242376490	0.001383	2.859
			MLMM	2	242376490	0.002105	2.677
		PMSnpB953	BLINK	3	278207076	0.001773	2.751
			MLMM	3	278207076	0.002655	2.576
		PMSnpB274	BLINK	1	266066748	0.002041	2.69
			MLMM	1	266066748	0.003028	2.519
		PMSnpB1026	BLINK	3	299049623	0.002151	2.667
			MLMM	3	299049623	0.003182	2.497
		PMSnpB1582	BLINK	5	154293023	0.002175	2.663
			MLMM	5	154293023	0.003215	2.493
		PMSnpB1977	BLINK	7	31652840	0.002799	2.553
			MLMM	7	31652840	0.004071	2.39
		PMSnpB584	BLINK	2	105218787	0.002892	2.539
			MLMM	2	105218787	0.004198	2.377
		PMSnp1655	BLINK	5	57686015	0.003246	2.489
			MLMM	5	57686015	0.004678	2.33
		PMSnp892	BLINK	3	14887346	0.003307	2.481
			MLMM	3	14887346	0.004761	2.322
		PMSnpB486	BLINK	2	46418733	0.003835	2.416
		PMSnpB1428	BLINK	5	99341486	0.00399	2.399
**21**	**SLC**	PMSnpB1046	BLINK	4	2335729	0.000255	3.593
			MLMM	4	2335729	0.000358	3.446
		PMSnp223	BLINK	1	176576809	0.000296	3.529
			MLMM	1	176576809	0.000413	3.384
		PMSnpB275	BLINK	1	266217584	0.000378	3.423
			MLMM	1	266217584	0.000522	3.282
		PMSnp225	BLINK	1	177680520	0.000485	3.314
			MLMM	1	177680520	0.000664	3.178
		PMSnpB1076	BLINK	4	13968214	0.000847	3.072
			MLMM	4	13968214	0.001133	2.946
		PMSnpB48	BLINK	1	28367195	0.001564	2.806
			MLMM	1	28367195	0.00204	2.69
		PMSnpB699	BLINK	2	240901231	0.00214	2.669

			MLMM	2	240901231	0.002756	2.56
		PMSnpB764	BLINK	3	11066983	0.002731	2.564
			MLMM	3	11066983	0.003482	2.458
		PMSnp885	BLINK	3	12499337	0.003958	2.402
			MLMM	3	12499337	0.00497	2.304
		PMSnpB1413	BLINK	5	93526975	0.004106	2.387
		PMSnp999	BLINK	3	73327925	0.004698	2.328
		PMSnpB844	BLINK	3	65928346	0.004709	2.327
**22**	**SVI1AA**	PMSnp1755	BLINK	5	107579664	0.000974	3.011
			MLMM	5	107579664	0.000017	4.78
		PMSnpB394	BLINK	2	15812931	0.001362	2.866
			MLMM	2	15812931	0.000075	4.12
		PMSnp1435	BLINK	4	79201633	0.001889	2.724
			MLMM	4	79201633	0.000136	3.868
		PMSnpB490	BLINK	2	48142589	0.002332	2.632
			MLMM	2	48142589	0.000328	3.484
		PMSnp2473	BLINK	7	89618943	0.002534	2.596
			MLMM	7	89618943	0.000549	3.26
		PMSnpB844	BLINK	3	65928346	0.003419	2.466
			MLMM	3	65928346	0.002451	2.611
		PMSnpB470	BLINK	2	44592445	0.003607	2.443
			MLMM	2	44592445	0.003108	2.508
		PMSnp578	BLINK	2	58312350	0.002747	2.561
		PMSnp1489	BLINK	4	132541695	0.003035	2.518
		PMSnpB408	BLINK	2	21500078	0.004106	2.387
		PMSnp1867	BLINK	5	144963046	0.004681	2.33
		PMSnp1505	BLINK	4	141674641	0.004782	2.32
		PMSnpB1047	MLMM	4	2360127	0.000334	3.476
		PMSnpB1379	MLMM	5	82505894	0.002581	2.588
		PMSnp2325	MLMM	7	20593751	0.004223	2.374
		PMSnpB1582	MLMM	5	154293023	0.004992	2.302
**23**	**SVI1AAR**	PMSnpB394	BLINK	2	15812931	0.000464	3.333
			MLMM	2	15812931	0.000025	4.6
		PMSnp1435	BLINK	4	79201633	0.001094	2.961
			MLMM	4	79201633	0.000086	4.07
		PMSnpB470	BLINK	2	44592445	0.001349	2.87
			MLMM	2	44592445	0.001035	2.985
		PMSnp1755	BLINK	5	107579664	0.00191	2.719
		PMSnpB408	BLINK	2	21500078	0.001056	2.977
		PMSnp578	BLINK	2	58312350	0.002883	2.54
		PMSnpB750	BLINK	3	6572955	0.003088	2.51
		PMSnp486	BLINK	2	15631126	0.003208	2.494
		PMSnp1489	BLINK	4	132541695	0.003712	2.43
		PMSnpB2111	BLINK	7	152636920	0.003747	2.426
		PMSnp1505	BLINK	4	141674641	0.004705	2.327
		PMSnpB490	MLMM	2	48142589	0.001121	2.95

		PMSnpB1047	MLMM	4	2360127	0.001317	2.88
		PMSnpB1611	MLMM	5	158614523	0.002691	2.57
		PMSnp2473	MLMM	7	89618943	0.00299	2.524
		PMSnpB1379	MLMM	5	82505894	0.003348	2.475
		PMSnpB1443	MLMM	5	111588861	0.003453	2.462
		PMSnpB268	MLMM	1	263211231	0.003948	2.404
		PMSnpB1456	MLMM	5	116241510	0.004464	2.35
		PMSnp1340	MLMM	4	30943673	0.004655	2.332
**24**	**SVI1C**	PMSnpB699	BLINK	2	240901231	0.000144	3.843
			MLMM	2	240901231	0.000031	4.5
		PMSnp1449	BLINK	4	97274250	0.000171	3.767
			MLMM	4	97274250	0.00006	4.22
		PMSnp2169	BLINK	6	201271649	0.003928	2.406
			MLMM	6	201271649	0.001636	2.786
		PMSnpB544	BLINK	2	71210193	0.001585	2.8
		PMSnp2205	BLINK	6	216095825	0.001606	2.794
		PMSnpB586	BLINK	2	108551940	0.001645	2.784
		PMSnpB844	BLINK	3	65928346	0.002014	2.696
		PMSnpB1172	BLINK	4	50614031	0.003132	2.504
		PMSnp472	BLINK	2	12660776	0.003426	2.465
		PMSnpB1097	BLINK	4	21151837	0.003491	2.457
		PMSnpB1356	BLINK	5	76356415	0.004027	2.395
		PMSnpB504	BLINK	2	53348531	0.004103	2.387
		PMSnpB2051	BLINK	7	103354573	0.004122	2.385
		PMSnpB955	MLMM	3	278704801	0.001561	2.806
		PMSnp576	MLMM	2	57313796	0.002546	2.594
		PMSnp2205	MLMM	6	216095825	0.003167	2.499
		PMSnpB114	MLMM	1	82568883	0.003829	2.417
		PMSnp2189	MLMM	6	210323969	0.003938	2.405
		PMSnpB937	MLMM	3	258208116	0.004759	2.322
		PMSnpB110	MLMM	1	76659805	0.004764	2.322
**25**	**SV12AA**	PMSnp2473	BLINK	7	89618943	0.000229	3.641
			MLMM	7	89618943	0.000031	4.5
		PMSnpB394	BLINK	2	15812931	0.000298	3.526
			MLMM	2	15812931	0.000042	4.38
		PMSnp1489	BLINK	4	132541695	0.000535	3.272
			MLMM	4	132541695	0.000751	3.125
		PMSnp578	BLINK	2	58312350	0.000661	3.18
			MLMM	2	58312350	0.001903	2.72
		PMSnpB470	BLINK	2	44592445	0.002005	2.698
			MLMM	2	44592445	0.002795	2.554
		PMSnp563	BLINK	2	46269689	0.003319	2.479
			MLMM	2	46269689	0.000856	3.068
		PMSnpB844	BLINK	3	65928346	0.004596	2.338
			MLMM	6	229236652	0.004441	2.352
		PMSnpB408	BLINK	2	21500078	0.000652	3.186

		PMSnp155	BLINK	1	118546611	0.001377	2.861
		PMSnp1505	BLINK	4	141674641	0.001818	2.74
		PMSnp2175	BLINK	6	204200353	0.002961	2.529
		PMSnpB2111	BLINK	7	152636920	0.003207	2.494
		PMSnpB556	BLINK	2	78445711	0.003621	2.441
		PMSnpB1156	BLINK	4	44044781	0.004604	2.337
		PMSnpB556	MLMM	2	78445711	0.000589	3.23
		PMSnp1435	MLMM	4	79201633	0.00288	2.541
		PMSnpB1047	MLMM	4	2360127	0.00289	2.539
		PMSnp2169	MLMM	6	201271649	0.003482	2.458
		PMSnp1755	MLMM	5	107579664	0.003498	2.456
		PMSnpB532	MLMM	2	66322133	0.003774	2.423
		PMSnpB1611	MLMM	5	158614523	0.004133	2.384
		PMSnp855	MLMM	2	242376490	0.004392	2.357
		PMSnpB1844	MLMM	6	229236652	0.004441	2.352
**26**	**SVI2AAR**	PMSnpB394	BLINK	2	15812931	0.000101	3.996
			MLMM	2	15812931	0.000036	4.44
		PMSnp1435	BLINK	4	79201633	0.000772	3.112
			MLMM	4	79201633	0.000265	3.576
		PMSnp1505	BLINK	4	141674641	0.003178	2.498
			MLMM	4	141674641	0.00277	2.557
		PMSnpB470	BLINK	2	44592445	0.003632	2.44
			MLMM	2	44592445	0.002878	2.541
		PMSnpB863	BLINK	3	157525175	0.004294	2.367
			MLMM	3	157525175	0.000559	3.253
		PMSnpB408	BLINK	2	21500078	0.000202	3.694
		PMSnpB2111	BLINK	7	152636920	0.002142	2.669
		PMSnpB1156	BLINK	4	44044781	0.003175	2.498
		PMSnpB844	BLINK	3	65928346	0.003692	2.433
		PMSnp578	BLINK	2	58312350	0.004882	2.311
		PMSnpB1611	MLMM	5	158614523	0.001159	2.936
		PMSnp563	MLMM	2	46269689	0.001285	2.891
		PMSnpB395	MLMM	2	17150912	0.001778	2.75
		PMSnp2473	MLMM	7	89618943	0.002116	2.674
		PMSnpB1363	MLMM	5	78147411	0.00276	2.559
		PMSnp2169	MLMM	6	201271649	0.003297	2.482
		PMSnpB1776	MLMM	6	119751831	0.003644	2.438
		PMSnpB490	MLMM	2	48142589	0.004087	2.389
**27**	**SVI2C**	PMSnpB2056	BLINK	7	105879125	0.000515	3.289
			MLMM	7	105879125	0.000566	3.247
		PMSnp225	BLINK	1	177680520	0.001555	2.808
			MLMM	1	177680520	0.001687	2.773
		PMSnpB1046	BLINK	4	2335729	0.001844	2.734
			MLMM	4	2335729	0.001996	2.7
		PMSnpB95	BLINK	1	68291958	0.002311	2.636
			MLMM	1	68291958	0.002495	2.603

	PMSnpB1166	BLINK	4	46294843	0.004354	2.361
		MLMM	4	46294843	0.004663	2.331
	PMSnpB2051	BLINK	7	103354573	0.004367	2.36
		MLMM	7	103354573	0.004676	2.33

Supplementary Table S4: Genes located within a 2 Mb range of significant SNPs related to seed longevity and vigor.

Sl. No.	SNP	Gene	Function/Description
**1**	PMSnpB2077	Pgl_GLEAN_10014447	Putative leucine-rich repeat receptor-like
**2**	PMSnpB2049	Pgl_GLEAN_10030023	Dof zinc finger protein DOF2.4-like
		Pgl_GLEAN_10030031	Expansin
		Pgl_GLEAN_10030037	GRF-type domain-containing protein
**3**	PMSnpB2046	Pgl_GLEAN_10030023	Dof zinc finger protein DOF2.4-like
		Pgl_GLEAN_10030031	Expansin
		Pgl_GLEAN_10030037	GRF-type domain-containing protein
**4**	PMSnpB2018	Pgl_GLEAN_10014673	LEA_2 domain-containing protein
**5**	PMSnpB1977	Pgl_GLEAN_10024337	Bromo domain-containing protein
		Pgl_GLEAN_10024342	WD_REPEATS_REGION domain-containing
		Pgl_GLEAN_10024350	Glycosyltransferase
		Pgl_GLEAN_10013924	Protein FAR1-RELATED SEQUENCE
		Pgl_GLEAN_10013912	Peroxidase
**6**	PMSnpB1952	Pgl_GLEAN_10012866	Peroxidase
		Pgl_GLEAN_10012867	Threonyl-tRNA synthetase
		Pgl_GLEAN_10012869	Lipase_GDSL domain-containing
**7**	PMSnpB1885	Pgl_GLEAN_10007407	NAC domain-containing protein
		Pgl_GLEAN_10007427	WRKY domain-containing protein
		Pgl_GLEAN_10011159	Peroxidase
		Pgl_GLEAN_10011162	AP2/ERF domain-containing protein
		Pgl_GLEAN_10011163	F-box domain-containing protein
		Pgl_GLEAN_10011168	Peroxidase
**8**	PMSnpB1649	Pgl_GLEAN_10007193	Glycosyltransferase
**9**	PMSnpB1648	Pgl_GLEAN_10007193	Glycosyltransferase
**10**	PMSnpB1611	Pgl_GLEAN_10023123	BTB/POZ domain-containing protein
**11**	PMSnpB1608	Pgl_GLEAN_10037757	TF-B3 domain-containing protein
		Pgl_GLEAN_10037771	Phospholipase D
		Pgl_GLEAN_10037790	F-box domain-containing protein
		Pgl_GLEAN_10037823	WD_REPEATS_REGION domain-containing
		Pgl_GLEAN_10037833	Xyloglucan endotransglucosylase/hydrolase
		Pgl_GLEAN_10037845	Skp1 domain-containing protein
		Pgl_GLEAN_10023123	BTB/POZ domain-containing protein
**12**	PMSnpB1606	Pgl_GLEAN_10037753	NAC-A/B domain-containing protein
		Pgl_GLEAN_10037756	Peroxidase
		Pgl_GLEAN_10037757	TF-B3 domain-containing protein
		Pgl_GLEAN_10037771	Phospholipase D
		Pgl_GLEAN_10037790	F-box domain-containing protein
		Pgl_GLEAN_10037823	WD_REPEATS_REGION domain-containing
		Pgl_GLEAN_10037830	Kinesin motor domain-containing
		Pgl_GLEAN_10037833	Xyloglucan endotransglucosylase/hydrolase
		Pgl_GLEAN_10037845	Skp1 domain-containing protein
		Pgl_GLEAN_10023123	BTB/POZ domain-containing protein

**13**	PMSnpB1582	Pgl_GLEAN_10037604	Beta-amylase
		Pgl_GLEAN_10037642	Glutathione transferase
		Pgl_GLEAN_10037658	NAC domain-containing protein
		Pgl_GLEAN_10037675	Glyceraldehyde-3-phosphate dehydrogenase
		Pgl_GLEAN_10037690	Bromo domain-containing protein
**14**	PMSnpB1556	Pgl_GLEAN_10002158	Bidirectional sugar transporter SWEET
		Pgl_GLEAN_10030742	AP2/ERF domain-containing protein
		Pgl_GLEAN_10030730	3-ketoacyl-CoA synthase
		Pgl_GLEAN_10030711	Lipoxygenase
		Pgl_GLEAN_10030684	Peroxidase
		Pgl_GLEAN_10030683	Glutathione transferase
		Pgl_GLEAN_10030656	WD_REPEATS_REGION domain-containing
**15**	PMSnpB1515	Pgl_GLEAN_10006520	HVA22-like protein
		Pgl_GLEAN_10023435	3-ketoacyl-CoA synthase
		Pgl_GLEAN_10023398	Xyloglucan endotransglucosylase/hydrolase
**16**	PMSnpB1514	Pgl_GLEAN_10006520	HVA22-like protein
		Pgl_GLEAN_10023435	3-ketoacyl-CoA
		Pgl_GLEAN_10023398	Xyloglucan endotransglucosylase/hydrolase
**17**	PMSnpB1456	Pgl_GLEAN_10034350	Glucose-6-phosphate 1-dehydrogenase
**18**	PMSnpB1441	Pgl_GLEAN_10002230	Peroxidase
**19**	PMSnpB1439	Pgl_GLEAN_10002230	Peroxidase
**20**	PMSnpB1428	Pgl_GLEAN_10028760	Peroxidase
		Pgl_GLEAN_10021788	Beta-amylase
		Pgl_GLEAN_10021758	AP2/ERF domain-containing protein
**21**	PMSnpB1363	Pgl_GLEAN_10038421	Peroxidase
		Pgl_GLEAN_10038433	Xyloglucan endotransglucosylase/hydrolase
**22**	PMSnpB1356	Pgl_GLEAN_10004643	Acyl-(Acyl-carrier-protein) desaturase,
		Pgl_GLEAN_10010129	F-box domain-containing protein
**23**	PMSnpB1295	Pgl_GLEAN_10006011	TF-B3 domain-containing protein
**24**	PMSnpB1259	Pgl_GLEAN_10023689	Malate dehydrogenase (NADP (+))
		Pgl_GLEAN_10001203	BTB domain-containing protein
**25**	PMSnpB1231	Pgl_GLEAN_10032011	Xyloglucan endotransglucosylase/hydrolase
		Pgl_GLEAN_10016075	2-alkenal reductase (NADP(+)-dependent)-like
**26**	PMSnpB1214	Pgl_GLEAN_10019636	ABC transporter F family member 1
**27**	PMSnpB1191	Pgl_GLEAN_10001201	Glycosyltransferase
		Pgl_GLEAN_10025263	Leucine-rich repeat receptor-like
		Pgl_GLEAN_10023945	PLAT domain-containing protein
**28**	PMSnpB1186	Pgl_GLEAN_10030884	F-box domain-containing protein
**29**	PMSnpB1172	Pgl_GLEAN_10032211	TF-B3 domain-containing protein
		Pgl_GLEAN_10032178	F-box domain-containing protein
**30**	PMSnpB1166	Pgl_GLEAN_10027443	WD_REPEATS_REGION domain-containing
		Pgl_GLEAN_10027434	Respiratory burst oxidase B
		Pgl_GLEAN_10001638	Auxin response factor 23
		Pgl_GLEAN_10003069	NAC domain-containing protein
**31**	PMSnpB1113	Pgl_GLEAN_10015189	BURP domain-containing protein
		Pgl_GLEAN_10015850	Peroxidase

**32**	PMSnpB1097	Pgl_GLEAN_10007872	Acyl carrier protein
		Pgl_GLEAN_10025482	Peroxidase
**33**	PMSnpB1076	Pgl_GLEAN_10003451	AP2/ERF domain-containing protein
		Pgl_GLEAN_10003453	BTB/POZ domain-containing protein
**34**	PMSnpB1047	Pgl_GLEAN_10019709	Kinesin motor domain-containing
		Pgl_GLEAN_10019721	BURP domain-containing protein
		Pgl_GLEAN_10004912	Mannan endo-1,4-beta-mannosidase
**35**	PMSnpB1046	Pgl_GLEAN_10019709	Kinesin motor domain-containing
		Pgl_GLEAN_10019721	BURP domain-containing protein
		Pgl_GLEAN_10004912	Mannan endo-1,4-beta-mannosidase
**36**	PMSnpB1026	Pgl_GLEAN_10018408	AP2-like ethylene-responsive transcription
		Pgl_GLEAN_10018506	Peroxidase
		Pgl_GLEAN_10031902	PI3K/PI4K domain-containing protein
**37**	PMSnpB990	Pgl_GLEAN_10018408	AP2-like ethylene-responsive transcription
		Pgl_GLEAN_10018506	Peroxidase
		Pgl_GLEAN_10031902	PI3K/PI4K domain-containing protein
**38**	PMSnpB979	Pgl_GLEAN_10020399	Pectin acetylesterase
		Pgl_GLEAN_10020387	Xyloglucan endotransglucosylase/hydrolase
**39**	PMSnpB977	Pgl_GLEAN_10020399	Pectin acetylesterase
		Pgl_GLEAN_10020387	Xyloglucan endotransglucosylase/hydrolase
**40**	PMSnpB971	Pgl_GLEAN_10023832	WRKY domain-containing protein
		Pgl_GLEAN_10004212	WD_REPEATS_REGION domain-containing
**41**	PMSnpB969	Pgl_GLEAN_10023832	WRKY domain-containing protein
		Pgl_GLEAN_10004212	WD_REPEATS_REGION domain-containing
**42**	PMSnpB937	Pgl_GLEAN_10035663	Auxin response factor
**43**	PMSnpB871	Pgl_GLEAN_10014283	SKP1-like protein
		Pgl_GLEAN_10038556	Glycosyltransferase
**44**	PMSnpB799	Pgl_GLEAN_10025339	LEA_2 domain-containing protein
		Pgl_GLEAN_10009590	AP2/ERF domain-containing protein
**45**	PMSnpB764	Pgl_GLEAN_10031586	WRKY domain-containing protein
		Pgl_GLEAN_10007773	AP2/ERF domain-containing protein
**46**	PMSnpB761	Pgl_GLEAN_10031604	Putative D-cysteine desulfhydrase
		Pgl_GLEAN_10031586	WRKY domain-containing protein
**47**	PMSnpB750	Pgl_GLEAN_10017335	DOG1 domain-containing protein
**48**	PMSnpB731	Pgl_GLEAN_10031083	BURP domain-containing protein
		Pgl_GLEAN_10031107	NAC domain-containing protein
		Pgl_GLEAN_10031111	ABC transporter domain-containing protein
		Pgl_GLEAN_10031112	Kinesin motor domain-containing protein
		Pgl_GLEAN_10031126	3-ketoacyl-CoA synthase
**49**	PMSnpB699	Pgl_GLEAN_10018178	Phospholipase D
**50**	PMSnpB594	Pgl_GLEAN_10010351	AP2/ERF domain-containing protein
**51**	PMSnpB586	Pgl_GLEAN_10015493	Xyloglucan endotransglucosylase/hydrolase
**52**	PMSnpB584	Pgl_GLEAN_10028178	Glycosyltransferase
		Pgl_GLEAN_10007608	HVA22-like protein
**53**	PMSnpB556	Pgl_GLEAN_10037096	Acyl-[acyl-carrier-protein] hydrolase
**54**	PMSnpB532	Pgl_GLEAN_10000783	F-box domain-containing protein

		Pgl_GLEAN_10010894	Mannan endo-1,4-beta-mannosidase
		Pgl_GLEAN_10010902	Glycosyltransferase
		Pgl_GLEAN_10010905	Lipoxygenase
**55**	PMSnpB504	Pgl_GLEAN_10011466	Glycosyltransferase
**56**	PMSnpB490	Pgl_GLEAN_10021992	3-ketoacyl-CoA synthase
		Pgl_GLEAN_10022009	Peptide transporter PTR3-A
**57**	PMSnpB486	Pgl_GLEAN_10016660	Auxin response factor
**58**	PMSnpB470	Pgl_GLEAN_10028712	ABC transporter domain-containing
**59**	PMSnpB462	Pgl_GLEAN_10034558	Malic enzyme
		Pgl_GLEAN_10001675	Auxin-responsive protein
**60**	PMSnpB436	Pgl_GLEAN_10011064	AP2/ERF domain-containing protein
**61**	PMSnpB395	Pgl_GLEAN_10013469	F-box domain-containing protein
		Pgl_GLEAN_10013466	UV-B-induced protein
**62**	PMSnpB394	Pgl_GLEAN_10023760	F-box domain-containing protein
		Pgl_GLEAN_10006432	WD_REPEATS_REGION domain-containing protein
		Pgl_GLEAN_10006436	Fip1 domain-containing protein
		Pgl_GLEAN_10013469	F-box domain-containing protein
		Pgl_GLEAN_10013466	UV-B-induced protein
**63**	PMSnpB374	Pgl_GLEAN_10013736	Dof-type domain-containing protein
		Pgl_GLEAN_10009714	Peroxidase
		Pgl_GLEAN_10006974	Alpha-1,4 glucan phosphorylase OS=Oryza
**64**	PMSnpB371	Pgl_GLEAN_10013736	Dof-type domain-containing protein
		Pgl_GLEAN_10009714	Peroxidase
		Pgl_GLEAN_10006974	Alpha-1,4 glucan phosphorylase
**65**	PMSnpB360	Pgl_GLEAN_10022839	Malate dehydrogenase
**66**	PMSnpB358	Pgl_GLEAN_10004672	NAC domain-containing protein
		Pgl_GLEAN_10022839	Malate dehydrogenase
**67**	PMSnpB337	Pgl_GLEAN_10023326	Germin-like protein
**68**	PMSnpB307	Pgl_GLEAN_10038305	Protein FAR1-RELATED SEQUENCE
		Pgl_GLEAN_10038309	LEA_2 domain-containing protein
		Pgl_GLEAN_10038318	HVA22-like protein
		Pgl_GLEAN_10038329	TF-B3 domain-containing protein
		Pgl_GLEAN_10038364	Germin-like protein
**69**	PMSnpB268	Pgl_GLEAN_10028577	Glycosyltransferase (Fragment)
		Pgl_GLEAN_10028571	Bidirectional sugar transporter SWEET
		Pgl_GLEAN_10011719	ABC transporter domain-containing
		Pgl_GLEAN_10003338	Auxin-responsive protein
**70**	PMSnpB264	Pgl_GLEAN_10004758	Peroxidase
		Pgl_GLEAN_10028600	Nudix hydrolase domain-containing
		Pgl_GLEAN_10028597	Lipase_GDSL domain-containing
		Pgl_GLEAN_10028577	Glycosyltransferase (Fragment)
		Pgl_GLEAN_10028571	Bidirectional sugar transporter SWEET
**71**	PMSnpB227	Pgl_GLEAN_10004180	Glycosyltransferase
		Pgl_GLEAN_10020097	Putative LRR receptor-like serine/threonine-protein
**72**	PMSnpB224	Pgl_GLEAN_10027890	Glycosyltransferase
		Pgl_GLEAN_10027896	Auxin-responsive protein

		Pgl_GLEAN_10027899	Malic enzyme
		Pgl_GLEAN_10027907	WRKY domain-containing protein
		Pgl_GLEAN_10004701	WD_REPEATS_REGION domain-containing protein
		Pgl_GLEAN_10019093	Glycosyltransferase
**73**	PMSnpB171	Pgl_GLEAN_10013543	NAC domain-containing protein
**74**	PMSnpB163	Pgl_GLEAN_10004328	Stromal 70 kDa heat shock-related
		Pgl_GLEAN_10023047	NAC domain-containing protein
		Pgl_GLEAN_10023061	Bidirectional sugar transporter SWEET
		Pgl_GLEAN_10023064	NAC domain-containing protein
**75**	PMSnpB137	Pgl_GLEAN_10020666	Putative peptide transporter
**76**	PMSnpB114	Pgl_GLEAN_10008638	Expansin
**77**	PMSnpB110	Pgl_GLEAN_10034696	NAC domain-containing protein
		Pgl_GLEAN_10034715	Glycosyltransferase
**78**	PMSnpB95	Pgl_GLEAN_10038138	Germin-like protein
**79**	PMSnpB40	Pgl_GLEAN_10017955	Glycosyltransferase
		Pgl_GLEAN_10028562	Phospholipase D
**80**	PMSnp2473	Pgl_GLEAN_10028306	Glycosyltransferase
		Pgl_GLEAN_10028273	F-box domain-containing protein
**81**	PMSnp2358	Pgl_GLEAN_10024342	WD_REPEATS_REGION domain-containing
		Pgl_GLEAN_10024350	Glycosyltransferase
		Pgl_GLEAN_10013924	Protein FAR1-RELATED SEQUENCE
		Pgl_GLEAN_10013912	Peroxidase
		Pgl_GLEAN_10013209	Glycosyltransferase
**82**	PMSnp2325	Pgl_GLEAN_10015794	F-box domain-containing protein
**83**	PMSnp2322	Pgl_GLEAN_10027314	CaM_binding domain-containing
**84**	PMSnp2287	Pgl_GLEAN_10022796	Peroxidase
		Pgl_GLEAN_10024293	10 kDa heat shock protein
**85**	PMSnp2268	Pgl_GLEAN_10007407	NAC domain-containing protein
		Pgl_GLEAN_10007427	WRKY domain-containing protein
		Pgl_GLEAN_10011159	Peroxidase
		Pgl_GLEAN_10011162	AP2/ERF domain-containing protein
		Pgl_GLEAN_10011163	F-box domain-containing protein
		Pgl_GLEAN_10011168	Peroxidase
		Pgl_GLEAN_10022942	Lipase_GDSL domain-containing
**86**	PMSnp2266	Pgl_GLEAN_10007407	NAC domain-containing protein
		Pgl_GLEAN_10007427	WRKY domain-containing protein
		Pgl_GLEAN_10011159	Peroxidase
		Pgl_GLEAN_10011162	AP2/ERF domain-containing protein
		Pgl_GLEAN_10011163	F-box domain-containing protein
		Pgl_GLEAN_10022942	Lipase_GDSL domain-containing
**87**	PMSnp2224	Pgl_GLEAN_10022643	Putative LRR receptor-like serine/threonine-protein
**88**	PMSnp2200	Pgl_GLEAN_10020730	ABC transporter domain-containing
**89**	PMSnp2189	Pgl_GLEAN_10008365	NAC domain-containing protein
**90**	PMSnp2175	Pgl_GLEAN_10030936	F-box domain-containing protein
		Pgl_GLEAN_10013283	Peroxidase
**91**	PMSnp2002	Pgl_GLEAN_10018882	Mannan endo-1,4-beta-mannosidase

		Pgl_GLEAN_10020911	WD_REPEATS_REGION domain-containing
**92**	PMSnp1943	Pgl_GLEAN_10007193	Glycosyltransferase
**93**	PMSnp1941	Pgl_GLEAN_10002302	Mitogen-activated protein kinase
		Pgl_GLEAN_10007193	Glycosyltransferase
**94**	PMSnp1867	Pgl_GLEAN_10031452	Histone H2B
**95**	PMSnp1755	Pgl_GLEAN_10027817	Phospholipase D
**96**	PMSnp1674	Pgl_GLEAN_10023967	NAC domain-containing protein
**97**	PMSnp1631	Pgl_GLEAN_10012531	Lipoxygenase OS=Setaria italica
		Pgl_GLEAN_10020287	Acyl-[acyl-carrier-protein] hydrolase
		Pgl_GLEAN_10020274	3-ketoacyl-CoA synthase
**98**	PMSnp1505	Pgl_GLEAN_10034303	Beta-galactosidase
		Pgl_GLEAN_10034282	Kinesin motor domain-containing protein
**99**	PMSnp1489	Pgl_GLEAN_10034303	Beta-galactosidase
**100**	PMSnp1449	Pgl_GLEAN_10032011	Xyloglucan endotransglucosylase/hydrolase
		Pgl_GLEAN_10016075	2-alkenal reductase (NADP(+)-dependent)-like
**101**	PMSnp1435	Pgl_GLEAN_10019636	ABC transporter F family member 1
**102**	PMSnp1416	Pgl_GLEAN_10014761	F-box protein SKIP19
		Pgl_GLEAN_10014764	F-box domain-containing protein
**103**	PMSnp1340	Pgl_GLEAN_10022454	F-box domain-containing protein
		Pgl_GLEAN_10022457	Auxin response factor
		Pgl_GLEAN_10022474	Bidirectional sugar transporter SWEET
**104**	PMSnp999	Pgl_GLEAN_10011587	LEA_2 domain-containing protein
		Pgl_GLEAN_10016751	F-box domain-containing protein
**105**	PMSnp959	Pgl_GLEAN_10025653	BTB domain-containing protein
**106**	PMSnp954	Pgl_GLEAN_10025855	BTB domain-containing protein
**107**	PMSnp892	Pgl_GLEAN_10018520	NAC domain-containing protein
		Pgl_GLEAN_10018528	Expansin
		Pgl_GLEAN_10018539	Kinesin motor domain-containing
		Pgl_GLEAN_10011541	Lipase_GDSL domain-containing
**108**	PMSnp885	Pgl_GLEAN_10012398	Glycosyltransferase
**109**	PMSnp855	Pgl_GLEAN_10018178	Phospholipase D
		Pgl_GLEAN_10018181	Lipase_GDSL domain-containing protein
		Pgl_GLEAN_10018073	Catalase
		Pgl_GLEAN_10003675	Kinesin motor domain-containing
		Pgl_GLEAN_10003681	Lipase_GDSL domain-containing
**110**	PMSnp854	Pgl_GLEAN_10018178	Phospholipase D
		Pgl_GLEAN_10018181	Lipase_GDSL domain-containing protein
		Pgl_GLEAN_10018073	Catalase
		Pgl_GLEAN_10003675	Kinesin motor domain-containing
**111**	PMSnp850	Pgl_GLEAN_10018178	Phospholipase D
		Pgl_GLEAN_10018181	Lipase_GDSL domain-containing protein
		Pgl_GLEAN_10018073	Catalase
**112**	PMSnp790	Pgl_GLEAN_10020460	Lipoxygenase
		Pgl_GLEAN_10017574	Kinesin motor domain-containing
**113**	PMSnp781	Pgl_GLEAN_10014609	F-box domain-containing protein
**114**	PMSnp578	Pgl_GLEAN_10012806	Peroxidase

		Pgl_GLEAN_10036164	BTB/POZ and MATH domain-containing
**115**	PMSnp576	Pgl_GLEAN_10012806	Peroxidase
**116**	PMSnp563	Pgl_GLEAN_10016660	Auxin response factor
**117**	PMSnp486	Pgl_GLEAN_10023760	F-box domain-containing protein
		Pgl_GLEAN_10006432	WD_REPEATS_REGION domain-containing protein
		Pgl_GLEAN_10006436	Fip1 domain-containing protein
**118**	PMSnp477	Pgl_GLEAN_10019532	Auxin-responsive protein
		Pgl_GLEAN_10019520	WRKY domain-containing protein
		Pgl_GLEAN_10002278	WD_REPEATS_REGION domain-containing
**119**	PMSnp472	Pgl_GLEAN_10023488	WD_REPEATS_REGION domain-containing
		Pgl_GLEAN_10019532	Auxin-responsive protein
		Pgl_GLEAN_10019520	WRKY domain-containing protein
**120**	PMSnp437	Pgl_GLEAN_10023326	Germin-like protein
		Pgl_GLEAN_10013179	Peroxidase
		Pgl_GLEAN_10013178	WRKY domain-containing protein
**121**	PMSnp436	Pgl_GLEAN_10023326	Germin-like protein
**122**	PMSnp357	Pgl_GLEAN_10021595	Pectin acetylesterase
		Pgl_GLEAN_10005264	F-box domain-containing protein
**123**	PMSnp317	Pgl_GLEAN_10006839	Lipase_GDSL domain-containing protein
		Pgl_GLEAN_10006837	ABC transporter domain-containing protein
		Pgl_GLEAN_10009457	Germin-like protein
**124**	PMSnp266	Pgl_GLEAN_10035688	Glutathione synthetase

Supplementary Table S5: Overview of MTAs linked to different seed longevity traits.

Sl. No.	SNP ID	No. of MTAs	Trait(s)
1	PMSnp1317	2	RLAARSLAAR
2	PMSnp1340	2	GRIAA,SVI1AAR
3	PMSnp1416	1	RLC
4	PMSnp1435	10	GAA,SVI1AAR,SV12AA,GAAR,GIAA,GIAAR,SVI1AA,GRIAA,GRIAAR,RLAAR
5	PMSnp1449	3	RLAAR,RLC,SVI1C
6	PMSnp1489	7	GAA,GIAA,GRIAA,SVI1AA,SVI1AAR,SV12AA
7	PMSnp1498	1	GRIAAR
8	PMSnp1505	11	GAA,GAAR,GC,GIAA,GIAAR,GIC,GRIAA,SVI1AA,SVI1AAR,SV12AA,SVI2AAR
9	PMSnp155	2	SDWAA,SV12AA
10	PMSnp1584	2	GRIC,MGTC
11	PMSnp1631	1	GRIAAR
12	PMSnp1653	1	SDWAAR
13	PMSnp1655	1	SLAAR
14	PMSnp1674	2	RLAAR,SLAA
15	PMSnp1755	9	GAA,GAAR,GIAAR,GRIAAR,RLAA,SLAA,SVI1AA,SVI1AAR,SV12AA,
16	PMSnp1867	1	SVI1AA
17	PMSnp1941	1	GIC
18	PMSnp1943	1	GC
19	PMSnp2002	3	GRIC,MGTAAR,MGTC
20	PMSnp2169	7	GAA,GC,GIAA,GIC,SVI1C,SV12AA,SVI2AAR
21	PMSnp2175	1	SV12AA
22	PMSnp2189	3	GC,GIC,SVI1C,
23	PMSnp2200	2	MGTAAR,MGTAAR
24	PMSnp2205	1	SVI1C
25	PMSnp2224	1	RLAA
26	PMSnp223	1	SLC
27	PMSnp225	3	SDWC,SVI2C,SLC
28	PMSnp2266	3	SDWAA,SDWAA,SLAA
29	PMSnp2268	1	RLC
30	PMSnp2287	1	MGTAA
31	PMSnp2322	1	RLAAR
32	PMSnp2325	3	SDWAA,SLAA,SVI1AA
33	PMSnp2330	3	GAA,GIAA,GRIAA
34	PMSnp2358	1	MGTAA
35	PMSnp2473	11	GAA,GAAR,GIAA,GIAAR,GRIAA,GRIAAR,SVI1AA,MGTAA,SVI1AAR,SV12AA,SVI2AAR,
36	PMSnp266	1	MGTAA
37	PMSnp317	1	GRIAA
38	PMSnp357	1	SDWC
39	PMSnp436	1	GRIAAR
40	PMSnp437	2	RLAAR,SDWAAR
41	PMSnp472	2	RLC,SVI1C

42	PMSnp477	1	GRIAA,
43	PMSnp486	3	GAAR,GIAAR,SVI1AAR,
44	PMSnp563	4	GAAR,GIAAR,SV12AA,SVI2AAR
45	PMSnp576	1	SVI1C,
46	PMSnp578	10	GAA,SVI2AAR,GAAR,GIAA,GIAAR,GRIAA,GRIAAR,SVI1AA,SVI1AAR,SV12AA
47	PMSnp620	1	SDWAA
48	PMSnp781	1	GC
49	PMSnp790	1	MGTC
50	PMSnp850	1	SDWAAR
51	PMSnp854	1	GC
52	PMSnp855	5	RLAA,SLAAR,RLAAR,SLAA,SV12AA,
53	PMSnp885	1	SLC,
54	PMSnp892	2	RLAAR,SLAAR
55	PMSnp90	2	SDWAAR,SDWC
56	PMSnp910	1	GRIAAR
57	PMSnp929	1	MGTC
58	PMSnp954	1	MGTAAR
59	PMSnp959	1	SDWAA
60	PMSnp999	1	SLC
61	PMSnpB1026	4	SLAAR
62	PMSnpB1046	3	SDWC,SLC,SVI2C
63	PMSnpB1047	6	GAA,GRIAAR,SLAA,SVI1AA,SVI1AAR,SV12AA
64	PMSnpB1073	1	SLAA
65	PMSnpB1076	3	GIAA,GRIAA,SLC
66	PMSnpB1097	1	SVI1C
67	PMSnpB110	1	SVI1C
68	PMSnpB1113	1	SDWAA
69	PMSnpB113	6	GAAR,GIAA,GIAAR,GIAAR,GRIAA,GRIAAR
70	PMSnpB114	1	SVI1C,
71	PMSnpB1156	2	SV12AA,SVI2AAR
72	PMSnpB1166	2	SDWC,SVI2C
73	PMSnpB1172	2	RLC,SVI1C
74	PMSnpB1186	3	GRIC,GRIC,MGTC
75	PMSnpB1191	1	SDWC
76	PMSnpB1207	1	SDWC
77	PMSnpB1214	1	GC
78	PMSnpB1219	1	GC
79	PMSnpB122	1	SDWAA
80	PMSnpB1231	1	RLC
81	PMSnpB1247	3	GAAR,GIAAR,GRIAAR
82	PMSnpB1259	1	RLC
83	PMSnpB1267	1	MGTC
84	PMSnpB1276	1	RLAAR
85	PMSnpB1295	1	RLAA
86	PMSnpB1322	1	MGTC
87	PMSnpB1356	1	SVI1C

88	PMSnpB1363	3	GRIAAR,MGTAAR,SVI2AAR
89	PMSnpB137	2	RLAA,SLAA
90	PMSnpB1379	2	SVI1AA,SVI1AAR
91	PMSnpB1413	1	SLC
92	PMSnpB1416	2	RLAAR,SLAA
93	PMSnpB1428	1	SLAAR
94	PMSnpB1439	2	GC,GIC
95	PMSnpB1441	2	RLAAR,SDWAAR
96	PMSnpB1443	1	SVI1AAR
97	PMSnpB145	1	MGTC
98	PMSnpB1456	1	SVI1AAR
99	PMSnpB1514	1	RLAAR
100	PMSnpB1515	1	RLAAR
101	PMSnpB1556	2	GIC,GRIC,MGTC
102	PMSnpB1582	2	SLAAR,SVI1AA
103	PMSnpB1606	1	SLAA
104	PMSnpB1608	1	SDWAAR
105	PMSnpB1611	5	GAAR,GIAAR,SVI1AAR,SV12AA,SVI2AAR
106	PMSnpB163	1	RLAA
107	PMSnpB1648	1	MGTAAR
108	PMSnpB1649	1	GRIAAR
109	PMSnpB171	4	RLAA,RLAAR,SDWAA,SLAA
110	PMSnpB1753	3	GIC,GRIC,MGTAAR
111	PMSnpB1776	2	SDWAAR,SVI2AAR
112	PMSnpB1799	1	RLC
113	PMSnpB1844	1	SV12AA
114	PMSnpB1885	1	RLAAR
115	PMSnpB1952	1	SLAA
116	PMSnpB1977	1	SLAAR
117	PMSnpB2005	1	MGTC
118	PMSnpB2018	4	GC,MGTC,GIC,GRIC
119	PMSnpB2046	1	MGTAA
120	PMSnpB2049	1	GRIC
121	PMSnpB2051	3	SVI1C,SVI2C,RLC
122	PMSnpB2056	3	SDWAA,SDWC,SVI2C
123	PMSnpB2077	1	SLAA
124	PMSnpB2111	9	GAA,GAAR,GIAA,GIAAR,GRIAA,GRIAAR,SVI1AAR,SV12AA,SVI2AAR
125	PMSnpB224	1	SLAA
126	PMSnpB227	1	RLAAR
127	PMSnpB264	1	MGTAAR
128	PMSnpB268	1	SVI1AAR
129	PMSnpB274	2	SLAA,SLAAR
130	PMSnpB275	1	SLC
131	PMSnpB307	3	SDWAAR,SLAAR,SLAA
132	PMSnpB337	1	GRIAAR
133	PMSnpB358	1	GAA

134	PMSnpB360	1	SDWAAR
135	PMSnpB371	1	SDWAAR
136	PMSnpB374	3	RLAAR,SLAAR,SLAA
137	PMSnpB394	14	GAA, GAAR, GIAAR, GIAA, GRIAA, GRIAAR, MGTAA, MGTAAR, SDWAA, SDWAAR, SVI1AA, SV12AA, SVI1AA, SVI2AAR
138	PMSnpB395	2	SDWAAR,SVI2AAR
139	PMSnpB40	2	MGTAA,RLAA
140	PMSnpB408	12	GAA,GAAR,GIAA,GIAAR,GRIAA,GRIAAR,SDWAA,SDWAAR,SVI1AA,SVI1AAR,SV12AA,SVI2AAR
141	PMSnpB420	1	SDWAAR
142	PMSnpB436	2	MGTAA,MGTAAR
143	PMSnpB462	1	GC
144	PMSnpB470	7	GAAR,GRIAAR,SVI1AA,GIAAR,SVI1AAR,SVI2AAR,SV12AA
145	PMSnpB48	1	SLC
146	PMSnpB486	1	SLAAR
147	PMSnpB490	8	GAA,GAAR,GRIAA,GIAA,GIAAR,GRIAAR,SVI1AA,SVI2AAR
148	PMSnpB504	1	SVI1C
149	PMSnpB532	6	GAA,GAAR,GIAA,GRIAA,GRIAAR,SV12AA
150	PMSnpB541	1	GRIAA
151	PMSnpB544	1	SVI1C
152	PMSnpB556	1	SV12AA
153	PMSnpB584	1	SLAAR
154	PMSnpB586	3	GC,GIC,SVI1C
155	PMSnpB594	1	SLAA
156	PMSnpB60	1	SDWC
157	PMSnpB648	1	SDWC
158	PMSnpB699	2	SLC,SVI1C
159	PMSnpB731	1	GRIAAR
160	PMSnpB750	3	GIAAR,GRIAAR,SVI1AAR
161	PMSnpB761	3	RLAA,SLAA,SLAAR
162	PMSnpB764	1	SLC
163	PMSnpB782	1	GIAA
164	PMSnpB784	6	GIAA,GIAAR,GRIAAR,GRIAAR,MGTAAR,MGTAAR
165	PMSnpB799	1	GRIAAR
166	PMSnpB844	11	GAA,GAAR,GIAA,GIAAR,GRIAA,GRIAAR,SLC,SVI1AA,SVI1C, SV12AA,SVI2AAR
167	PMSnpB863	3	GAAR,GIAAR,SVI2AAR
168	PMSnpB871	1	GIC
169	PMSnpB902	4	RLAA,RLAAR,SLAA,SLAAR
170	PMSnpB937	1	SVI1C
171	PMSnpB95	1	SVI2C
172	PMSnpB953	2	SLAA,SLAAR
173	PMSnpB955	3	GC,SVI1C,GIC
174	PMSnpB956	1	RLAAR
175	PMSnpB969	1	MGTAA
176	PMSnpB971	1	SLAA
177	PMSnpB977	2	SLAA,SLAAR

178	PMSnpB979	3	RLAAR,SLAA,SLAAR
179	PMSnpB988	1	RLAA
180	PMSnpB990	1	GC

Supplementary Table S6: Description of the genes illustrated in the circos plot.

Chr No	Gene_Id	Gene description	Abbrevation
chr7	Pgl_GLEAN_10030023	Dof zinc finger protein DOF2.4-like	DOF2.4
chr7	Pgl_GLEAN_10030031	Expansin	EXP
chr7	Pgl_GLEAN_10030037	GRF-type domain-containing protein	GRF
chr4	Pgl_GLEAN_10022454	F-box domain-containing protein	F-box
chr4	Pgl_GLEAN_10022457	Auxin response factor	ARF
chr4	Pgl_GLEAN_10022474	Bidirectional sugar transporter SWEET	SWEET
chr4	Pgl_GLEAN_10014761	F-box protein SKIP19	SKIP19
chr4	Pgl_GLEAN_10014764	F-box domain-containing protein	F-box
chr4	Pgl_GLEAN_10019636	ABC transporter F family member 1	ABC-F1
chr4	Pgl_GLEAN_10032011	Xyloglucan endotransglucosylase/hydrolase	XTH
chr4	Pgl_GLEAN_10016075	2-alkenal reductase (NADP(+)-dependent)-like	AER
chr4	Pgl_GLEAN_10034303	Beta-galactosidase	BGAL
chr4	Pgl_GLEAN_10034303	Beta-galactosidase (duplicate)	BGAL
chr4	Pgl_GLEAN_10034282	Kinesin motor domain-containing protein	Kinesin
chr5	Pgl_GLEAN_10012531	Lipoxygenase	LOX
chr5	Pgl_GLEAN_10020287	Acyl-[acyl-carrier-protein] hydrolase	ACPH
chr5	Pgl_GLEAN_10020274	3-ketoacyl-CoA synthase	KCS
chr5	Pgl_GLEAN_10023967	NAC domain-containing protein	NAC
chr5	Pgl_GLEAN_10027817	Phospholipase D	PLD
chr5	Pgl_GLEAN_10031452	Histone H2B	H2B
chr6	Pgl_GLEAN_10002302	Mitogen-activated protein kinase	MAPK
chr6	Pgl_GLEAN_10007193	Glycosyltransferase	GT
chr6	Pgl_GLEAN_10007193	Glycosyltransferase (duplicate)	GT
chr6	Pgl_GLEAN_10018882	Mannan endo-1,4-beta-mannosidase	MAN
chr6	Pgl_GLEAN_10020911	WD_REPEATS_REGION domain-containing	WD-repeat
chr6	Pgl_GLEAN_10030936	F-box domain-containing protein	F-box
chr6	Pgl_GLEAN_10013283	Peroxidase	PRX
chr6	Pgl_GLEAN_10008365	NAC domain-containing protein	NAC
chr6	Pgl_GLEAN_10020730	ABC transporter domain-containing	ABC
chr6	Pgl_GLEAN_10022643	Putative LRR receptor-like serine/threonine-protein	LRR
chr7	Pgl_GLEAN_10007407	NAC domain-containing protein	NAC
chr7	Pgl_GLEAN_10007427	WRKY domain-containing protein	WRKY
chr7	Pgl_GLEAN_10011159	Peroxidase	PRX
chr7	Pgl_GLEAN_10011162	AP2/ERF domain-containing protein	AP2/ERF
chr7	Pgl_GLEAN_10011163	F-box domain-containing protein	F-box
chr7	Pgl_GLEAN_10022942	Lipase_GDSL domain-containing	GDSL
chr7	Pgl_GLEAN_10007407	NAC domain-containing protein	NAC
chr7	Pgl_GLEAN_10007427	WRKY domain-containing protein	WRKY
chr7	Pgl_GLEAN_10011159	Peroxidase	PRX
chr7	Pgl_GLEAN_10011162	AP2/ERF domain-containing protein	AP2/ERF
chr7	Pgl_GLEAN_10011163	F-box domain-containing protein	F-box
chr7	Pgl_GLEAN_10011168	Peroxidase	PRX

chr7	Pgl_GLEAN_10022942	Lipase_GDSL domain-containing	GDSL
chr7	Pgl_GLEAN_10022796	Peroxidase	PRX
chr7	Pgl_GLEAN_10024293	10 kDa heat shock protein	HSP10
chr7	Pgl_GLEAN_10027314	CaM_binding domain-containing	CaM
chr7	Pgl_GLEAN_10015794	F-box domain-containing protein	F-box
chr7	Pgl_GLEAN_10024342	WD_REPEATS_REGION domain-containing	WD-repeat
chr7	Pgl_GLEAN_10024350	Glycosyltransferase	GT
chr7	Pgl_GLEAN_10013924	Protein FAR1-RELATED SEQUENCE	FAR1
chr7	Pgl_GLEAN_10013912	Peroxidase	PRX
chr7	Pgl_GLEAN_10013209	Glycosyltransferase	GT
chr7	Pgl_GLEAN_10028306	Glycosyltransferase	GT
chr7	Pgl_GLEAN_10028273	F-box domain-containing protein	F-box
chr1	Pgl_GLEAN_10035688	Glutathione synthetase	GSS
chr1	Pgl_GLEAN_10006839	Lipase_GDSL domain-containing protein	GDSL
chr1	Pgl_GLEAN_10006837	ABC transporter domain-containing protein	ABC
chr1	Pgl_GLEAN_10009457	Germin-like protein	GLP
chr1	Pgl_GLEAN_10021595	Pectin acetylesterase	PAE
chr1	Pgl_GLEAN_10005264	F-box domain-containing protein	F-box
chr2	Pgl_GLEAN_10023326	Germin-like protein	GLP
chr2	Pgl_GLEAN_10023326	Germin-like protein	GLP
chr2	Pgl_GLEAN_10013179	Peroxidase	PRX
chr2	Pgl_GLEAN_10013178	WRKY domain-containing protein	WRKY
chr2	Pgl_GLEAN_10023488	WD_REPEATS_REGION domain-containing	WD-repeat
chr2	Pgl_GLEAN_10019532	Auxin-responsive protein	ARP
chr2	Pgl_GLEAN_10019520	WRKY domain-containing protein	WRKY
chr2	Pgl_GLEAN_10019532	Auxin-responsive protein	ARP
chr2	Pgl_GLEAN_10019520	WRKY domain-containing protein	WRKY
chr2	Pgl_GLEAN_10002278	WD_REPEATS_REGION domain-containing	WD-repeat
chr2	Pgl_GLEAN_10023760	F-box domain-containing protein	F-box
chr2	Pgl_GLEAN_10006432	WD_REPEATS_REGION domain-containing protein	WD-repeat
chr2	Pgl_GLEAN_10006436	Fip1 domain-containing protein	Fip1
chr2	Pgl_GLEAN_10016660	Auxin response factor	ARF
chr2	Pgl_GLEAN_10012806	Peroxidase	PRX
chr2	Pgl_GLEAN_10012806	Peroxidase	PRX
chr2	Pgl_GLEAN_10036164	BTB/POZ and MATH domain-containing	BTB/MATH
chr2	Pgl_GLEAN_10014609	F-box domain-containing protein	F-box
chr2	Pgl_GLEAN_10020460	Lipoxygenase	LOX
chr2	Pgl_GLEAN_10017574	Kinesin motor domain-containing	Kinesin
chr2	Pgl_GLEAN_10018178	Phospholipase D	PLD
chr2	Pgl_GLEAN_10018181	Lipase_GDSL domain-containing protein	GDSL
chr2	Pgl_GLEAN_10018073	Catalase	CAT
chr2	Pgl_GLEAN_10018178	Phospholipase D	PLD
chr2	Pgl_GLEAN_10018181	Lipase_GDSL domain-containing protein	GDSL
chr2	Pgl_GLEAN_10018073	Catalase	CAT
chr2	Pgl_GLEAN_10003675	Kinesin motor domain-containing	Kinesin
chr2	Pgl_GLEAN_10018178	Phospholipase D	PLD

chr2	Pgl_GLEAN_10018181	Lipase_GDSL domain-containing protein	GDSL
chr2	Pgl_GLEAN_10018073	Catalase	CAT
chr2	Pgl_GLEAN_10003675	Kinesin motor domain-containing	Kinesin
chr2	Pgl_GLEAN_10003681	Lipase_GDSL domain-containing	GDSL
chr3	Pgl_GLEAN_10012398	Glycosyltransferase	GT
chr3	Pgl_GLEAN_10018520	NAC domain-containing protein	NAC
chr3	Pgl_GLEAN_10018528	Expansin	EXP
chr3	Pgl_GLEAN_10018539	Kinesin motor domain-containing	Kinesin
chr3	Pgl_GLEAN_10011541	Lipase_GDSL domain-containing	GDSL
chr3	Pgl_GLEAN_10025855	BTB domain-containing protein	BTB
chr3	Pgl_GLEAN_10025653	BTB domain-containing protein	BTB
chr3	Pgl_GLEAN_10016751	F-box domain-containing protein	F-box
chr3	Pgl_GLEAN_10018408	AP2-like ethylene-responsive transcription	AP2-ethylene
chr3	Pgl_GLEAN_10018506	Peroxidase	PRX
chr3	Pgl_GLEAN_10031902	PI3K/PI4K domain-containing protein	PI3K/PI4K
chr3	Pgl_GLEAN_10019709	Kinesin motor domain-containing	Kinesin
chr4	Pgl_GLEAN_10019721	BURP domain-containing protein	BURP
chr4	Pgl_GLEAN_10004912	Mannan endo-1,4-beta-mannosidase	MAN
chr4	Pgl_GLEAN_10019709	Kinesin motor domain-containing	Kinesin
chr4	Pgl_GLEAN_10019721	BURP domain-containing protein	BURP
chr4	Pgl_GLEAN_10004912	Mannan endo-1,4-beta-mannosidase	MAN
chr4	Pgl_GLEAN_10003451	AP2/ERF domain-containing protein	AP2/ERF
chr4	Pgl_GLEAN_10003453	BTB/POZ domain-containing protein	BTB/POZ
chr4	Pgl_GLEAN_10007872	Acyl carrier protein	ACP
chr4	Pgl_GLEAN_10025482	Peroxidase	PRX
chr3	Pgl_GLEAN_10034696	NAC domain-containing protein	NAC
chr3	Pgl_GLEAN_10034715	Glycosyltransferase	GT
chr4	Pgl_GLEAN_10015189	BURP domain-containing protein	BURP
chr4	Pgl_GLEAN_10015850	Peroxidase	PRX
chr1	Pgl_GLEAN_10008638	Expansin	EXP
chr4	Pgl_GLEAN_10027443	WD_REPEATS_REGION domain-containing	WD-repeat
chr4	Pgl_GLEAN_10027434	Respiratory burst oxidase B	RBOB
chr4	Pgl_GLEAN_10001638	Auxin response factor 23	ARF23
chr4	Pgl_GLEAN_10003069	NAC domain-containing protein	NAC
chr4	Pgl_GLEAN_10032211	TF-B3 domain-containing protein	TF-B3
chr4	Pgl_GLEAN_10032178	F-box domain-containing protein	F-box
chr4	Pgl_GLEAN_10030884	F-box domain-containing protein	F-box
chr4	Pgl_GLEAN_10001201	Glycosyltransferase	GT
chr4	Pgl_GLEAN_10025263	Leucine-rich repeat receptor-like	LRR
chr4	Pgl_GLEAN_10023945	PLAT domain-containing protein	PLAT
chr4	Pgl_GLEAN_10019636	ABC transporter F family member 1	ABC-F1
chr4	Pgl_GLEAN_10032011	Xyloglucan endotransglucosylase/hydrolase	XTH
chr4	Pgl_GLEAN_10016075	2-alkenal reductase (NADP(+)-dependent)-like	AER
chr4	Pgl_GLEAN_10023689	Malate dehydrogenase (NADP (+))	MDH
chr4	Pgl_GLEAN_10001203	BTB domain-containing protein	BTB
chr4	Pgl_GLEAN_10006011	TF-B3 domain-containing protein	TF-B3

chr5	Pgl_GLEAN_10004643	Acyl-(Acyl-carrier-protein) desaturase	ACD
chr5	Pgl_GLEAN_10010129	F-box domain-containing protein	F-box
chr5	Pgl_GLEAN_10038421	Peroxidase	PRX
chr5	Pgl_GLEAN_10038433	Xyloglucan endotransglucosylase/hydrolase	XTH
chr1	Pgl_GLEAN_10020666	Putative peptide transporter	PPT
chr5	Pgl_GLEAN_10028760	Peroxidase	PRX
chr5	Pgl_GLEAN_10021788	Beta-amylase	BAM
chr5	Pgl_GLEAN_10021758	AP2/ERF domain-containing protein	AP2/ERF
chr5	Pgl_GLEAN_10002230	Peroxidase	PRX
chr5	Pgl_GLEAN_10002230	Peroxidase	PRX
chr5	Pgl_GLEAN_10034350	Glucose-6-phosphate 1-dehydrogenase	G6PD
chr5	Pgl_GLEAN_10006520	HVA22-like protein	HVA22
chr5	Pgl_GLEAN_10023435	3-ketoacyl-CoA	K3AC
chr5	Pgl_GLEAN_10023398	Xyloglucan endotransglucosylase/hydrolase	XTH
chr5	Pgl_GLEAN_10006520	HVA22-like protein	HVA22
chr5	Pgl_GLEAN_10023435	3-ketoacyl-CoA synthase	KCS
chr5	Pgl_GLEAN_10023398	Xyloglucan endotransglucosylase/hydrolase	XTH
chr5	Pgl_GLEAN_10002158	Bidirectional sugar transporter SWEET	SWEET
chr5	Pgl_GLEAN_10030742	AP2/ERF domain-containing protein	AP2/ERF
chr5	Pgl_GLEAN_10030730	3-ketoacyl-CoA synthase	KCS
chr5	Pgl_GLEAN_10030711	Lipoxygenase	LOX
chr5	Pgl_GLEAN_10030684	Peroxidase	PRX
chr5	Pgl_GLEAN_10030683	Glutathione transferase	GST
chr5	Pgl_GLEAN_10030656	WD_REPEATS_REGION domain-containing	WD-repeat
chr5	Pgl_GLEAN_10037604	Beta-amylase	BAM
chr5	Pgl_GLEAN_10037642	Glutathione transferase	GST
chr5	Pgl_GLEAN_10037658	NAC domain-containing protein	NAC
chr5	Pgl_GLEAN_10037675	Glyceraldehyde-3-phosphate dehydrogenase	G3PDH
chr5	Pgl_GLEAN_10037690	Bromo domain-containing protein	Bromo
chr5	Pgl_GLEAN_10037753	NAC-A/B domain-containing protein	NAC-A/B
chr5	Pgl_GLEAN_10037756	Peroxidase	PRX
chr5	Pgl_GLEAN_10037757	TF-B3 domain-containing protein	TF-B3
chr5	Pgl_GLEAN_10037771	Phospholipase D	PLD
chr5	Pgl_GLEAN_10037790	F-box domain-containing protein	F-box
chr5	Pgl_GLEAN_10037823	WD_REPEATS_REGION domain-containing	WD-repeat
chr5	Pgl_GLEAN_10037830	Kinesin motor domain-containing	Kinesin
chr5	Pgl_GLEAN_10037833	Xyloglucan endotransglucosylase/hydrolase	XTH
chr5	Pgl_GLEAN_10037845	Skp1 domain-containing protein	Skp1
chr5	Pgl_GLEAN_10023123	BTB/POZ domain-containing protein	BTB/POZ
chr5	Pgl_GLEAN_10037757	TF-B3 domain-containing protein	TF-B3
chr5	Pgl_GLEAN_10037771	Phospholipase D	PLD
chr5	Pgl_GLEAN_10037790	F-box domain-containing protein	F-box
chr5	Pgl_GLEAN_10037823	WD_REPEATS_REGION domain-containing	WD-repeat
chr5	Pgl_GLEAN_10037833	Xyloglucan endotransglucosylase/hydrolase	XTH
chr5	Pgl_GLEAN_10037845	Skp1 domain-containing protein	Skp1
chr5	Pgl_GLEAN_10023123	BTB/POZ domain-containing protein	BTB/POZ

chr5	Pgl_GLEAN_10023123	BTB/POZ domain-containing protein	BTB/POZ
chr1	Pgl_GLEAN_10004328	Stromal 70 kDa heat shock-related	HSP70
chr1	Pgl_GLEAN_10023047	NAC domain-containing protein	NAC
chr1	Pgl_GLEAN_10023061	Bidirectional sugar transporter SWEET	SWEET
chr1	Pgl_GLEAN_10023064	NAC domain-containing protein	NAC
chr6	Pgl_GLEAN_10007193	Glycosyltransferase	GT
chr6	Pgl_GLEAN_10007193	Glycosyltransferase	GT
chr1	Pgl_GLEAN_10013543	NAC domain-containing protein	NAC
chr7	Pgl_GLEAN_10007407	NAC domain-containing protein	NAC
chr7	Pgl_GLEAN_10007427	WRKY domain-containing protein	WRKY
chr7	Pgl_GLEAN_10011159	Peroxidase	PRX
chr7	Pgl_GLEAN_10011162	AP2/ERF domain-containing protein	AP2/ERF
chr7	Pgl_GLEAN_10011163	F-box domain-containing protein	F-box
chr7	Pgl_GLEAN_10011168	Peroxidase	PRX
chr7	Pgl_GLEAN_10012866	Peroxidase	PRX
chr7	Pgl_GLEAN_10012867	Threonyl-tRNA synthetase	Thr-tRNAsynth
chr7	Pgl_GLEAN_10012869	Lipase_GDSL domain-containing	GDSL
chr7	Pgl_GLEAN_10024337	Bromo domain-containing protein	Bromo
chr7	Pgl_GLEAN_10024342	WD_REPEATS_REGION domain-containing	WD-repeat
chr7	Pgl_GLEAN_10024350	Glycosyltransferase	GT
chr7	Pgl_GLEAN_10013924	Protein FAR1-RELATED SEQUENCE	FAR1
chr7	Pgl_GLEAN_10013912	Peroxidase	PRX
chr7	Pgl_GLEAN_10014673	LEA_2 domain-containing protein	LEA2
chr7	Pgl_GLEAN_10030023	Dof zinc finger protein DOF2.4-like	DOF2.4
chr7	Pgl_GLEAN_10030031	Expansin	EXP
chr7	Pgl_GLEAN_10030037	GRF-type domain-containing protein	GRF
chr7	Pgl_GLEAN_10014447	Putative leucine-rich repeat receptor-like	LRR
chr1	Pgl_GLEAN_10027890	Glycosyltransferase	GT
chr1	Pgl_GLEAN_10027896	Auxin-responsive protein	Auxin-RP
chr1	Pgl_GLEAN_10027899	Malic enzyme	ME
chr1	Pgl_GLEAN_10027907	WRKY domain-containing protein	WRKY
chr1	Pgl_GLEAN_10004701	WD_REPEATS_REGION domain-containing protein	WD-repeat
chr1	Pgl_GLEAN_10019093	Glycosyltransferase	GT
chr1	Pgl_GLEAN_10004180	Glycosyltransferase	GT
chr1	Pgl_GLEAN_10020097	Putative LRR receptor-like serine/threonine-protein	LRR-ST
chr1	Pgl_GLEAN_10004758	Peroxidase	PRX
chr1	Pgl_GLEAN_10028600	Nudix hydrolase domain-containing	Nudix
chr1	Pgl_GLEAN_10028597	Lipase_GDSL domain-containing	GDSL
chr1	Pgl_GLEAN_10028577	Glycosyltransferase (Fragment)	GT-F
chr1	Pgl_GLEAN_10028571	Bidirectional sugar transporter SWEET	SWEET
chr1	Pgl_GLEAN_10028577	Glycosyltransferase (Fragment)	GT-F
chr1	Pgl_GLEAN_10028571	Bidirectional sugar transporter SWEET	SWEET
chr1	Pgl_GLEAN_10011719	ABC transporter domain-containing	ABC
chr1	Pgl_GLEAN_10003338	Auxin-responsive protein	Auxin-RP
chr1	Pgl_GLEAN_10038305	Protein FAR1-RELATED SEQUENCE	FAR1
chr1	Pgl_GLEAN_10038309	LEA_2 domain-containing protein	LEA2

chr1	Pgl_GLEAN_10038318	HVA22-like protein	HVA22
chr1	Pgl_GLEAN_10038329	TF-B3 domain-containing protein	TF-B3
chr1	Pgl_GLEAN_10038364	Germin-like protein	Germin
chr2	Pgl_GLEAN_10023326	Germin-like protein	Germin
chr2	Pgl_GLEAN_10004672	NAC domain-containing protein	NAC
chr2	Pgl_GLEAN_10022839	Malate dehydrogenase	MDH
chr2	Pgl_GLEAN_10022839	Malate dehydrogenase	MDH
chr2	Pgl_GLEAN_10013736	Dof-type domain-containing protein	DOF
chr2	Pgl_GLEAN_10009714	Peroxidase	PRX
chr2	Pgl_GLEAN_10006974	Alpha-1,4 glucan phosphorylase	AGP
chr2	Pgl_GLEAN_10013736	Dof-type domain-containing protein	DOF
chr2	Pgl_GLEAN_10009714	Peroxidase	PRX
chr2	Pgl_GLEAN_10006974	Alpha-1,4 glucan phosphorylase	AGP
chr2	Pgl_GLEAN_10023760	F-box domain-containing protein	F-box
chr2	Pgl_GLEAN_10006432	WD_REPEATS_REGION domain-containing protein	WD-repeat
chr2	Pgl_GLEAN_10006436	Fip1 domain-containing protein	Fip1
chr2	Pgl_GLEAN_10013469	F-box domain-containing protein	F-box
chr2	Pgl_GLEAN_10013466	UV-B-induced protein	UV-B
chr2	Pgl_GLEAN_10013469	F-box domain-containing protein	F-box
chr2	Pgl_GLEAN_10013466	UV-B-induced protein	UV-B
chr1	Pgl_GLEAN_10017955	Glycosyltransferase	GT
chr1	Pgl_GLEAN_10028562	Phospholipase D	PLD
chr2	Pgl_GLEAN_10011064	AP2/ERF domain-containing protein	AP2/ERF
chr2	Pgl_GLEAN_10034558	Malic enzyme	ME
chr2	Pgl_GLEAN_10001675	Auxin-responsive protein	Auxin-RP
chr2	Pgl_GLEAN_10028712	ABC transporter domain-containing	ABC
chr2	Pgl_GLEAN_10016660	Auxin response factor	ARF
chr2	Pgl_GLEAN_10021992	3-ketoacyl-CoA synthase	KCS
chr2	Pgl_GLEAN_10022009	Peptide transporter PTR3-A	PTR3
chr2	Pgl_GLEAN_10011466	Glycosyltransferase	GT
chr2	Pgl_GLEAN_10000783	F-box domain-containing protein	F-box
chr2	Pgl_GLEAN_10010894	Mannan endo-1,4-beta-mannosidase	ManE
chr2	Pgl_GLEAN_10010902	Glycosyltransferase	GT
chr2	Pgl_GLEAN_10010905	Lipoxygenase	LOX
chr2	Pgl_GLEAN_10037096	Acyl-[acyl-carrier-protein] hydrolase	ACP-hydrolase
chr2	Pgl_GLEAN_10028178	Glycosyltransferase	GT
chr2	Pgl_GLEAN_10007608	HVA22-like protein	HVA22
chr2	Pgl_GLEAN_10015493	Xyloglucan endotransglucosylase/hydrolase	XTH
chr2	Pgl_GLEAN_10010351	AP2/ERF domain-containing protein	AP2/ERF
chr2	Pgl_GLEAN_10018178	Phospholipase D	PLD
chr3	Pgl_GLEAN_10031083	BURP domain-containing protein	BURP
chr3	Pgl_GLEAN_10031107	NAC domain-containing protein	NAC
chr3	Pgl_GLEAN_10031111	ABC transporter domain-containing protein	ABC
chr3	Pgl_GLEAN_10031112	Kinesin motor domain-containing protein	Kinesin
chr3	Pgl_GLEAN_10031126	3-ketoacyl-CoA synthase	KCS
chr3	Pgl_GLEAN_10017335	DOG1 domain-containing protein	DOG1

chr3	Pgl_GLEAN_10031604	Putative D-cysteine desulfhydrase	D-cysteine
chr3	Pgl_GLEAN_10031586	WRKY domain-containing protein	WRKY
chr3	Pgl_GLEAN_10031586	WRKY domain-containing protein	WRKY
chr3	Pgl_GLEAN_10007773	AP2/ERF domain-containing protein	AP2/ERF
chr3	Pgl_GLEAN_10025339	LEA_2 domain-containing protein	LEA2
chr3	Pgl_GLEAN_10009590	AP2/ERF domain-containing protein	AP2/ERF
chr3	Pgl_GLEAN_10014283	SKP1-like protein	SKP1
chr3	Pgl_GLEAN_10038556	Glycosyltransferase	GT
chr3	Pgl_GLEAN_10035663	Auxin response factor	ARF
chr1	Pgl_GLEAN_10038138	Germin-like protein	Germin
chr3	Pgl_GLEAN_10023832	WRKY domain-containing protein	WRKY
chr3	Pgl_GLEAN_10004212	WD_REPEATS_REGION domain-containing	WD-repeat
chr3	Pgl_GLEAN_10023832	WRKY domain-containing protein	WRKY
chr3	Pgl_GLEAN_10004212	WD_REPEATS_REGION domain-containing	WD-repeat
chr3	Pgl_GLEAN_10020399	Pectin acetylesterase	PAE
chr3	Pgl_GLEAN_10020387	Xyloglucan endotransglucosylase/hydrolase	XTH
chr3	Pgl_GLEAN_10020399	Pectin acetylesterase	PAE
chr3	Pgl_GLEAN_10020387	Xyloglucan endotransglucosylase/hydrolase	XTH
chr3	Pgl_GLEAN_10018408	AP2-like ethylene-responsive transcription	AP2-like
chr3	Pgl_GLEAN_10018506	Peroxidase	PRX
chr3	Pgl_GLEAN_10031902	PI3K/PI4K domain-containing protein	PI3K/PI4K
